# Architecture and functions of a multipartite genome of the methylotrophic bacterium *Paracoccus aminophilus* JCM 7686, containing primary and secondary chromids

**DOI:** 10.1186/1471-2164-15-124

**Published:** 2014-02-12

**Authors:** Lukasz Dziewit, Jakub Czarnecki, Daniel Wibberg, Monika Radlinska, Paulina Mrozek, Michal Szymczak, Andreas Schlüter, Alfred Pühler, Dariusz Bartosik

**Affiliations:** 1 Department of Bacterial Genetics, Institute of Microbiology, Faculty of Biology, University of Warsaw, Miecznikowa 1, 02-096 Warsaw, Poland; 2 Institute for Genome Research and Systems Biology, Center for Biotechnology, Bielefeld University, D-33594 Bielefeld, Germany; 3 Department of Virology, Institute of Microbiology, Faculty of Biology, University of Warsaw, Miecznikowa 1, 02-096 Warsaw, Poland

**Keywords:** *Paracoccus aminophilus* JCM 7686, Genome, Chromid, Plasmid, Mobile genetic element, Bacteriophage

## Abstract

**Background:**

*Paracoccus aminophilus* JCM 7686 is a methylotrophic α-*Proteobacterium* capable of utilizing reduced one-carbon compounds as sole carbon and energy source for growth, including toxic *N,N*-dimethylformamide, formamide, methanol, and methylamines, which are widely used in the industry. *P. aminophilus* JCM 7686, as many other *Paracoccus* spp., possesses a genome representing a multipartite structure, in which the genomic information is split between various replicons, including chromids, essential plasmid-like replicons, with properties of both chromosomes and plasmids. In this study, whole-genome sequencing and functional genomics approaches were applied to investigate *P. aminophilus* genome information.

**Results:**

The *P. aminophilus* JCM 7686 genome has a multipartite structure, composed of a single circular chromosome and eight additional replicons ranging in size between 5.6 and 438.1 kb. Functional analyses revealed that two of the replicons, pAMI5 and pAMI6, are essential for host viability, therefore they should be considered as chromids. Both replicons carry housekeeping genes, e.g. responsible for *de novo* NAD biosynthesis and ammonium transport. Other mobile genetic elements have also been identified, including 20 insertion sequences, 4 transposons and 10 prophage regions, one of which represents a novel, functional serine recombinase-encoding bacteriophage, ϕPam-6. Moreover, *in silico* analyses allowed us to predict the transcription regulatory network of the JCM 7686 strain, as well as components of the stress response, recombination, repair and methylation machineries. Finally, comparative genomic analyses revealed that *P. aminophilus* JCM 7686 has a relatively distant relationship to other representatives of the genus *Paracoccus*.

**Conclusions:**

*P. aminophilus* genome exploration provided insights into the overall structure and functions of the genome, with a special focus on the chromids. Based on the obtained results we propose the classification of bacterial chromids into two types: “primary” chromids, which are indispensable for host viability and “secondary” chromids, which are essential, but only under some environmental conditions and which were probably formed quite recently in the course of evolution. Detailed genome investigation and its functional analysis, makes *P. aminophilus* JCM 7686 a suitable reference strain for the genus *Paracoccus*. Moreover, this study has increased knowledge on overall genome structure and composition of members within the class *Alphaproteobacteria*.

## Background

The genus *Paracoccus* (*Alphaproteobacteria*) currently comprises 40 recognized and validly named species, isolated from different environments in various geographical locations. Members of this genus exhibit a broad range of metabolic flexibility, especially in respiratory processes, e.g. employing nitrate, nitrite, nitrous oxide and nitric oxide as alternative electron acceptors in denitrification and the ability to use one-carbon (C1) compounds (e.g. methanol, methylamine) as electron donors to respiratory chains [1]. Moreover, *Paracoccus* spp. as facultative chemolithoautotrophs may utilize reduced sulfur compounds (e.g. thiocyanate, thiosulfate or elemental sulfur), molecular hydrogen and Fe(II) as energy sources [2-4]. *Paracoccus* spp. are also able to use a broad range of organic compounds as their sole source of carbon and energy, including pollutants such as acetone, dichloromethane, formamide, *N,N*-dimethylformamide (DMF) and methylamine [3,5]. Another common feature of *Paracoccus* spp. is methylotrophy, defined as the ability to utilize reduced C1 carbon substrates containing no carbon-carbon bonds (including methane, methanol, methylated amines, halogenated methanes and methylated sulfur species) as their sole source of carbon and energy for growth.

Having a versatile metabolism, *Paracoccus* spp. play an important role in biogeochemical cycles and they have also been successfully employed in the biotreatment of contaminated environments, e.g. bioremediation of soils contaminated with polycyclic aromatic hydrocarbons (PAHs) using *Paracoccus* sp. HPD-2 [6].

Although *Paracoccus* spp. constitute an interesting and metabolically versatile group of bacteria with substantial biotechnological potential, little is known about the content and organization of their genomes. Only one complete genome of *Paracoccus denitrificans* PD1222 has been deposited in the NCBI database ([GenBank:CP000489], [GenBank:CP000490] and [GenBank:CP000491]). This genome is composed of two chromosomes (ChI – 2.9 Mb and ChII – 1.7 Mb) and a single megaplasmid (plasmid 1) of 653 kb. Four *Paracoccus* spp. genome sequencing projects (*Paracoccus* sp. TRP [7], *Paracoccus denitrificans* SD1 [8], *Paracoccus* sp. N5 and *P. zeaxanthinifaciens* ATCC 21588) are currently in progress.

Much more is known about mobile genetic elements (MGEs) of *Paracoccus* spp. Baj and colleagues (2000) [9] demonstrated that bacteria belonging to this genus usually harbor at least one plasmid (in most cases a mega-sized replicon, exceeding 100 kb). Several multireplicon strains carrying 4 or more plasmids were identified, including *P. aminophilus* JCM 7686. Our group has already obtained the sequences of 18 plasmids, ranging in size from 2.7 to 40 kb, that originate from different *Paracoccus* spp.: *P. methylutens* DM12 (2 plasmids) [10], *P. pantotrophus* DSM 11072 (1 plasmid) [11] and four carotenoid producers, *P. aestuarii* DSM 19484 (5 plasmids) ([GenBank:JQ041633], [GenBank:JQ065021], [GenBank:JQ066766], [GenBank:JQ684025], [GenBank:JQ796370]), *P. haeundaensis* LMG P-21903 (2 plasmids) ([GenBank:JQ066767], [GenBank:JQ684024]), *P. marcusii* DSM 11574 (5 plasmids) ([GenBank: KC542384], [GenBank:KC561053], [GenBank:KC561054], [GenBank:KC561055], [GenBank:JQ796371) and *P. marcusii* OS22 (3 plasmids) ([GenBank:JQ664550], [GenBank:JQ678602], [GenBank:JQ684023]). We have also sequenced the basic replicons of three other plasmids from *P. alcaliphilus* JCM 7364 (pALC1) and *P. versutus* UW1 (pTAV1 and pTAV3) [12-14]. Moreover, complex analyses of 25 *Paracoccus* spp. strains using trap plasmid systems have led to the identification and characterization of (i) 48 insertion sequences (ISs), (ii) a composite transposon Tn*6097* carrying genetic modules involved in the arginine deiminase pathway and daunorubicin/doxorubicin resistance, (iii) 3 non-composite transposons of the Tn*3* family, (iv) a transposable genomic island Tn*Ppa1* (45 kb) and (v) several transposable modules (TMos) generated by a single copy of the IS*1380* family insertion sequence [10,15-20]. The findings outlined above suggest that horizontal gene transfer (HGT) events occur frequently in *Paracoccus* spp. genomes, which may explain their metabolic flexibility.

Among *Paracoccus* species, *P. aminophilus* JCM 7686 is of particular interest since it is a methylotrophic bacterium capable of utilizing several toxic C1 compounds, including *N,N*-dimethylformamide, formamide as well as tri-, di- and monomethylamine, which are widely used in the chemical industry [21]. *P. aminophilus* JCM 7686 carries eight indigenous, extrachromosomal replicons (pAMI1 to pAMI8) ranging in size from 5.6 kb to approximately 440 kb. Our previous analyses, focused on the three smallest plasmids, pAMI3 (5.6 kb), pAMI2 (18.6 kb) and pAMI7 (20.5 kb), revealed the presence of (i) novel types of plasmid-encoded maintenance systems [22,23], (ii) a type II restriction-modification module with NcoI specificity [24] and (iii) genes crucial for the first step in the degradation of DMF [25].

In the present study, the genome of *P. aminophilus* JCM 7686 was completely sequenced and analyzed. An in-depth exploration of this genome sequence, followed by functional analyses, provided considerable insights into its overall architecture, as well as the functions of particular replicons. Moreover, in this study, the first inducible *Paracoccus* phage was identified.

## Results and discussion

### Sequencing and general features of the *P. aminophilus* JCM 7686 genome

A 454-pyrosequencing run for the *P. aminophilus* JCM7686 genomic DNA yielded 598348 shotgun and 8-kb-long paired-end reads with a total number of 225,950,536 bp that were assembled into 17 scaffolds. The scaffolds consisted of 429 large (> 500 nucleotides) and 129 small (100–500 nucleotides) contigs. The gaps in the chromosome and plasmids were closed by a PCR-based approach followed by sequencing of the corresponding amplicons. Subsequently, the genome was re-sequenced (for the quality check) applying the Illumina HiScanSQ Genome Analyzer.

**Figure 1:**
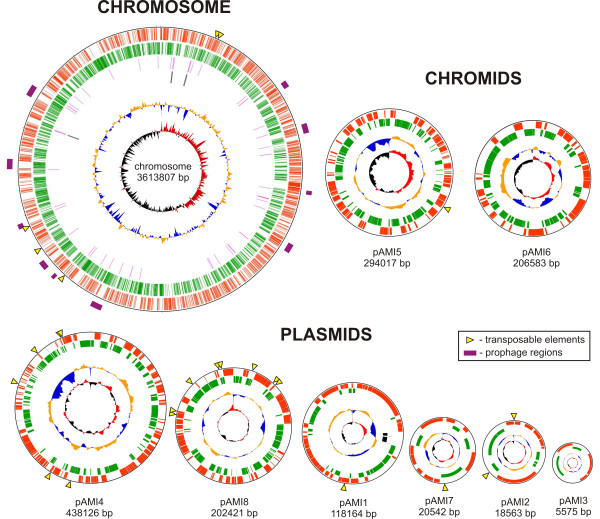
**Circular representations of the *P. aminophilus *JCM 7686 genome.** Circles displayed (from the outside): (i) predicted CDSs transcribed in the clockwise direction, (ii) predicted CDSs transcribed in the counterclockwise direction, (iii) the position of tRNA genes, (iv) the position of rRNA genes, (v) the GC percent deviation, (vi) GC skew (G + C/G-C). The tRNA and rRNA genes are present only within the chromosome and plasmid pAMI1. Yellow arrow heads indicate transposable elements. Violet lines represents the prophage-like regions. Circles are drawn not in scale.

The genome of *P. aminophilus* JCM 7686 is composed of a single circular chromosome of 3,613,807 bp and eight circular plasmids: pAMI1 (118,164 bp), pAMI2 (18,563 bp), pAMI3 (5575 bp), pAMI4 (438,126 bp), pAMI5 (294,017 bp), pAMI6 (206,583 bp), pAMI7 (20,542 bp) and pAMI8 (202,421 bp) (Figure 1). Thus, the total size of the genome is 4,917,798 bp. The overall GC content of the chromosome is 63.4%, which is consistent with other sequenced *Paracoccus* genomes. The GC contents of the JCM 7686 plasmids ranges between 57.4% (pAMI7) and 64.2% (pAMI4) (Table 1).

**Table 1:** **General features of the *P. aminophilus *** **JCM 7686 genome**

**General features**	**Chromosome**	**pAMI1**	**pAMI2**	**pAMI3**	**pAMI4**	**pAMI5**	**pAMI6**	**pAMI7**	**pAMI8**
Size (bp)	3,613,807	118,164	18,563	5575	438,126	294,017	206,583	20,542	202,421
GC content (%)	63.4	63.3	62	60.8	64.2	62.8	63.9	57.4	62.3
Coding density (%)	90.2	87.5	79.1	72	88.1	88.5	92.8	83.6	89.2
Number of ORFs	3416	99	19	7	375	264	171	19	203
Number of tRNA genes	56	3	0	0	0	0	0	0	0
Number of 16S-23S-5S rRNA operons	3	1	0	0	0	0	0	0	0
Phage regions	9	0	0	0	0	0	0	0	0
GTA regions	1	0	0	0	0	0	0	0	0
Transposases (including truncated)	25	1	4	0	20	4	0	1	17
Complete non-composite transposons	0	1	0	0	0	0	0	1	2
Complete insertion sequences	5	0	2	0	6	1	0	0	6

The genome of JCM 7686 possesses 59 tRNAs and four clusters of 5S, 16S and 23S rRNA genes, which are located within the chromosome and plasmid pAMI1 (Table 1). This strain can produce tRNAs for all 20 amino acids, and genes encoding all the aminoacyl-tRNA synthetases are present within its chromosome. Moreover, a single tRNA for selenocyteine (tRNA-SeC) was also identified.

The JCM 7686 genome contains 4573 putative coding sequences (CDSs): 3416 in the chromosome and 1157 in the plasmids (Table 1). The coding density within the chromosome is 90.2%, while within the plasmids it varies from 72% (pAMI3) to 92.8% (pAMI6) (Table 1). We could assign putative biological functions to 3506 CDSs (76.6%), while 1069 CDSs (23.4%) were annotated as encoding hypothetical proteins of unknown function.

Predicted *P. aminophilus* proteins were functionally categorized and the proportions in each COG category were calculated. A total of 1550 (33.9%) predicted proteins were described as involved in the overall cellular metabolism, being assigned to COG functional categories C, G, E, F, H, I or Q. It appeared that more than 40% of the genes encoding proteins involved in metabolic processes are located within extrachromosomal elements, mainly pAMI1, pAMI4, pAMI5 and pAMI6 (426 genes in total). This finding suggests that these four replicons perform an important role (Additional file 1).

The most abundant class of proteins comprises those involved in amino acid transport and metabolism (COG category E). A total of 576 (12.6%) CDSs were assigned to this category, of which 191 occur within the aforementioned replicons: pAMI1 (41 genes), pAMI4 (65), pAMI5 (52) and pAMI6 (31) (Additional file 1).

### Transcription regulatory network

Within the JCM 7686 genome, we identified 12 genes (7 in the chromosome, 3 in pAMI4, 1 in pAMI1 and 1 in pAMI5) encoding predicted Sigma factors: (i) FecI-like σ^19^ – required for the uptake of iron (5 copies, JCM7686_1690, JCM7686_1812, JCM7686_pAMI1p074, JCM7686_pAMI4p059 and JCM7686_pAMI5p016), (ii) RpoE-like σ^24^ – enabling the expression of genes involved in the heat shock response, as well as extracytoplasmic transport (4 copies, JCM7686_2748, JCM7686_2865, JCM7686_3466 and JCM7686_pAMI4p292), (iii) RpoH-like σ^32^ – involved in the heat shock and other stress responses (JCM7686_2477), (iv) RpoN-like σ^54^ – involved in nitrogen metabolism (JCM7686_pAMI4p216) and (v) RpoD-like σ^70^ – regulating gene expression during exponential growth (JCM7686_2204). Genes equivalent to *rpoF* (σ^28^) and *rpoS* (σ^38^), responsible for the expression of flagellar genes and genes in the stationary phase of growth, respectively, were not detected in this genome. In addition, we identified anti-sigma (JCM7686_3151) and anti-anti-sigma (JCM7686_3150) factors within the JCM 7686 chromosome.

The presence of eleven alternative sigma factors (of four types – σ^19^, σ^24^, σ^32^ and σ^54^), enables global changes in gene expression and permits alteration of the overall cellular metabolism in response to changing environmental conditions [26]. Thus, this feature indirectly reflects the metabolic flexibility and adaptive abilities of the strain JCM 7686. It is noteworthy that three of five *P. aminophilus fecI* (σ^19^) genes were identified within plasmids, which suggests an important role for these replicons in iron transport and metabolism. Moreover, the only *rpoN* gene copy was carried by a plasmid, pAMI4. It was previously demonstrated that RpoN, besides regulating genes involved in nitrogen metabolism, enables the transcription of a wide range of other genes encoding proteins participating in the regulation of virulence-related factors, as well as in amino acid, carbohydrate and organic acid synthesis, utilization and transport [26]. This indicates that pAMI4 may be highly significant in overall gene regulation in the strain JCM 7686.

Global regulators, which have the ability to regulate operons that belong to various metabolic pathways, are another basic component of transcription regulatory networks in bacteria. It has been shown that the expression of 51% of the *E. coli* genes is under the control of only seven regulatory proteins: CRP, FNR, IHF, FIS, ArcA, NarL and Lrp [27]. Within the JCM 7686 genome we identified genes encoding 5 types of putative global gene regulators: (i) three CRP/FNR family transcriptional regulators (JCM7686_1106, JCM7686_3086, JCM7686_pAMI8p144), (ii) three FIS family transcriptional regulators (JCM7686_3395, JCM7686_pAMI4p202, JCM7686_pAMI5p202), (iii) a H-NS proteins (JCM7686_3426, JCM7686_pAMI8p115), (iv) a Lrp regulator (JCM7686_0212) and (v) integration host factor (IHF) subunits A (IhfA; JCM7686_1093) and B (IhfB; JCM7686_1288).

We also identified 217 genes encoding transcriptional regulators with predicted local specificity of action, probably limited to a single gene/gene cluster (Additional file 2). A quarter of the identified regulators were assigned to the LysR family – the most abundant type of transcriptional regulators in bacteria [28]. Interestingly, we identified genes encoding 8 regulators of the LuxR family, which are located within the chromosome and plasmids pAMI1, pAMI2, pAMI4 and pAMI8. The majority of LuxR-type proteins represents transcription activators, which specifically bind to *N*-acyl homoserine lactones (AHL; synthesized by a LuxI protein) that are secreted signaling molecules involved in quorum sensing in a variety of Gram-negative bacteria (e.g. [29]). We also identified CDSs for 3 putative *N*-acyl-*L*-homoserine lactone synthetases (LuxI-like proteins): two localized within the chromosome (JCM7686_2124, JCM7686_3181) and one in plasmid pAMI1 (JCM7686_pAMI1p026). The presence of *luxR* and *luxI* genes strongly suggests that quorum sensing plays a role in regulation of *P. aminophilus* gene expression.

Two-component systems are another important part of bacterial transcription regulatory networks present in *P. aminophilus*. Such systems are directly involved in sensing a cell’s external environment and signal transduction (e.g. [30]). Within the JCM 7686 genome we identified 23 pairs of genes encoding histidine protein kinases (HPKs) and phospho-aspartyl response regulators: 15 of them within the chromosome and others located on plasmids pAMI4, pAMI5, pAMI6 and pAMI8 (Additional file 3). Moreover, we identified 6 HPKs with unknown partner response regulators in the chromosome and pAMI6 (Additional file 3).

Among the *P. aminophilus* HPKs we found homologs of enzymes of well-described two-component phosphorelay systems involved in regulating (i) nitrogen assimilation – NtrB (JCM7686_0575 and NtrX (JCM7686_0577) [31], (ii) chemotaxis – CheA (JCM7686_1281) [32], (iii) phosphate homeostasis – PhoR (JCM7686_2063) [33], (iv) differentiation and cell cycle progression – CckA (JCM7686_2539) [34], (v) C4-dicarboxylate metabolism – DctB (JCM7686_2824) [35], (vi) expression of virulence factors – QseC (JCM7686_3369; JCM7686_pAMI5p117) [36], (vii) methanol and formaldehyde oxidation – FlhS (JCM7686_3383), (viii) several anaerobic processes and assimilation of CO_2_ and N_2_ – RegB (JCM7686_3423) [37], (ix) high affinity potassium-uptake – KdpD (JCM7686_pAMI4p337) [38] and (x) expression of the *tor* structural operon encoding the trimethylamine *N*-oxide reductase respiratory system in response to substrate availability – TorS (JCM7686_pAMI5p018) [39].

### Stress response

In the majority of cases, the processes underlying the global stress response of bacteria are dependent on alterations in gene expression, usually controlled at the transcriptional level by various sigma factors [40]. However, there are also several more specific stress response mechanisms.

A fundamental trigger of the cellular stress response is DNA damage. In this scenario, the bacterial defense mechanism relies on a conserved inducible pathway – the SOS response (e.g. [41]). The primary components of this pathway, RecA recombinase and the LexA repressor, are encoded within the JCM 7686 chromosome (JCM7686_2538 and JCM7686_0753, respectively).

Moreover, the SpoT/RelA, (p)ppGpp synthetase I (JCM7686_1948), involved in the bacterial stringent response (triggered by nutritional deprivation) was also identified. During the stringent response process, an accumulation of the signaling nucleotides pppGpp and ppGpp occurs, which causes dramatic alterations in gene expression [42]. An important role in this process is also played by the chromosomally-encoded RelE toxin of the *relBE* systems, which, as mRNA-cleaving enzymes, globally inhibits translation during amino acid starvation [43]. Within the *P. aminophilus* genome we identified 11 toxin-antitoxin (TA) pairs representing the families *relBE/parDE* (5 TA systems), *phd-doc* (1), *ccdAB* (1), *hipAB* (2), and 2 hybrid systems (*vapC-phd*). The majority of the identified TA modules were localized within the plasmids (Additional file 4), and they most probably perform replicon stabilization functions. Only a single TA system of the *hipAB* family was located on the chromosome.

Among the most important factors in the maintenance of cellular fitness under changing environmental conditions are molecular chaperones. They are involved in various processes in bacterial cells, such as assisting the folding of newly synthesized proteins, protein secretion, preventing the aggregation of proteins under heat shock conditions, and repairing proteins that have been damaged or misfolded due to stress conditions (e.g. [44]). Within the *P. aminophilus* genome we identified 35 putative molecular chaperones, the majority of which (32) are encoded by the chromosome. These include heat and cold shock proteins, proteases, disulfide bond chaperones and protein-export chaperones (Additional file 5). We also identified a major RNA chaperone, Hfq, which is a key player in small RNA (sRNA)-mediated regulation of target mRNAs, that promotes sRNA-mRNA base pairing to permit rapid adaptive responses [45]. Surprisingly, we did not identify homologs of the bacterial HtpG (Hsp90) protein, which is responsible for heat or chemical shock responses. This is unusual because genes encoding HtpG proteins are present in the majority of bacterial genomes [46].

### DNA recombination and repair

Since the integrity of the genomic DNA is fundamental to bacterial persistence, it is extremely important that the cell is equipped with sufficient DNA repair proteins to protect it against genetic damage [47]. Within the JCM 7686 genome we identified 59 genes encoding proteins predicted to be involved in various DNA repair pathways. The vast majority of them (47) are located within the chromosome. We performed a detailed characterization and classification of all genes encoding DNA repair-related proteins (summarized in Additional file 6). Comparison of the DNA repair genes of *P. aminophilus* with those of two well defined species, *Escherichia coli* (*Gammaproteobacteria*) and *Caulobacter crescentus* (*Alphaproteobacteria*), revealed the presence of 53 and 58 orthologous genes, respectively (Additional file 6).

As in *C. crescentus*[47], the JCM 7686 genome contains no *mutH* and *dam* homologs, which implies that both strains use different proteins to recognize and repair DNA replication errors. *P. aminophilus*, like many other *Alphaproteobacteria* (including *C. crescentus*) also lacks the RecBCD module, but possesses the related system AddAB instead, which recognizes a 5-base Chi site (5′-AGCGG-3′; 17,218 such recognition sequences were identified within the JCM 7686 genome) [48]. Curiously, we also found that *P. aminophilus* does not encode a typical deoxyribodipyrimidine photolyase (Phr-like). These enzymes employ visible light as the energy source to monomerize pyrimidine dimers induced by UV irradiation [49], and orthologs are found within the *C. crescentus* genome. Instead of Phr, JCM 7686 encodes a non-related SplB-like DNA repair photolyase. It was shown that the SplB protein is a DNA repair enzyme responsible for the process of reversion of the thymine dimer, 5-thyminyl-5,6-dihydrothymine (spore photoproduct), formed during UV irradiation of *Bacillus subtilis* spores [50]. We hypothesize that this protein may function as the major photolyase of *P. aminophilus*, responsible for the photo-reactivation process.

Although the majority of the DNA repair proteins are found within the JCM 7686 chromosome, 12 genes encoding such proteins are present in three plasmids: pAMI4 (3 genes), pAMI5 (3) and pAMI8 (6). Among them is an *alkB* gene (carried by pAMI5), which encodes a highly conserved and usually chromosomally-encoded protein (AlkB), responsible for the repair of alkylation damage in DNA *via* an oxidative demethylation pathway [51]. The presence of this *alkB* gene within pAMI5 suggests that it may be an essential replicon.

### DNA methylation

Genes encoding predicted DNA methyltransferases (MTases), which may play important roles in the regulation of replication, gene expression and mismatch repair systems (e.g. [52]) were detected in the JCM 7686 genome. In addition to the previously described M.PamI [24], that is part of the type II restriction-modification system encoded by plasmid pAMI7, we identified seven chromosomally-encoded putative MTase genes. All of them seem to be orphan MTases, since they lack associated partner endonucleases.

The gene JCM7685_3079 encodes a CcrM (cell-cycle regulating MTase) homolog. Its ability to modify adenine residues in GANTC sequences was confirmed *in vivo* and *in vitro* (Additional file 7). The CcrM methyltransferases were shown to be essential for the viability of various *Alphaproteobacteria* (including *Caulobacter crescentus*, *Sinorhizobium meliloti*, *Agrobacterium tumefaciens* and *Brucella abortus*) and to play a crucial role in the regulation of bacterial cell division (e.g. [53]). To confirm the pivotal role of the JCM7686_3079 gene product in the *P. aminophilus* cell cycle, we attempted to disrupt this gene. However, this proved to be impossible, unless a wild-type copy was provided *in trans*, demonstrating that this CcrM homolog is essential for the viability of the host strain.

The other six genes encoding putative MTases are located within predicted prophage regions. Based on automatic methyltransferase prediction algorithms we allocated the following genes to different MTase classes: m^6^A MTases – JCM7686_1231, JCM7686_2255 and JCM7686_2934; m^4^C MTase – JCM7686_0815; m^5^C MTases – JCM7686_0772 and JCM7686_2655. Each of these predicted methylase genes was cloned into an expression vector and expressed in *E. coli*. The activity of the recombinant MTases was assessed and their sequence specificity determined using the endonuclease protection assay (according to [54]) (Additional files 8, 9, 10).

This analysis revealed that both m^5^C MTases have relaxed substrate specificity [partial protection of sequences CCNGG, CCWGG, AGCT, CCGG, GCN_7_GC and others (Additional file 8)], but they do not protect DNA against cleavage by R.PamI, encoded by the restriction-modification system of pAMI7. The m^4^C MTase of JCM 7686 recognizes and methylates YGGCCR sequences (Additional file 9). The three m^6^A MTases are highly similar (at least 95% aa sequence identity) and they methylate the sequence GANTC (Additional file 10). This is the same sequence specificity as assigned to the CcrM methylase (the main cell cycle regulator).

### Multireplicon structure of the JCM 7686 genome

As mentioned above, the genome of *P. aminophilus* JCM 7686 is composed of a single chromosome and eight smaller replicons (pAMI1-pAMI8) (Figure 1). Predicted plasmids constitute 26.5% of the genome and they carry 1158 CDSs (about 25% of all JCM 7686 CDSs), which means that a huge amount of genetic information is stored within these replicons. Among the JCM 7686 plasmids we distinguished 5 mega-sized replicons (118–438 kb), pAMI1, pAMI4, pAMI5, pAMI6 and pAMI8, all carrying genes conserved in bacterial chromosomes, including chromosomes I and II of *P. denitrificans* PD1222 (Additional file 11). Since size is not an infallible criterion for distinguishing plasmids from secondary chromosomes, functional analyses of the JCM 7686 replicons was undertaken.

To characterize the function of the mega-sized replicons in the cellular metabolism we constructed mini-derivatives, which were used to remove the native plasmids from the host cell by incompatibility (such analyses of the smallest plasmids pAMI2, pAMI3 and pAMI7 were performed previously [22,23,25]. The mini-derivatives were constructed by cloning DNA fragments containing the plasmid maintenance modules (including plasmid replication initiation modules), into the *E. coli*-specific, mobilizable, narrow-host-range vector pABW1 (ColE1-type *ori* of pMB1) [55]. Using the obtained mini-derivatives containing incompatibility determinants, we were able to remove plasmids pAMI1, pAMI4, pAMI6 and pAMI8 from JCM 7686 (Additional file 12). Interestingly, curing this strain of pAMI1 resulted in a change in colony morphology, which became irregular, flat and dry. We were unable to introduce the mini-derivative of pAMI5 into JCM 7686 cells. It was therefore impossible to remove pAMI5, which strongly suggests that this replicon might be essential to host viability.

**Figure 2:**
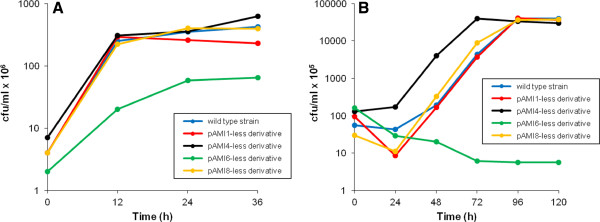
**The growth rates of pAMI1-, pAMI4-, pAMI6- and pAMI8-less derivatives of *P. aminophilus *JCM 7686.** Growth rates of the bacterial cultures were determined by viable cell counts (cfu).The strains [wild-type strain, pAMI1-less derivative, pAMI4-less derivative, pAMI6-less derivative and pAMI8-less derivative] were grown in 20 ml of liquid LB **(A)** or AC medium **(B)** with arabinose added as the sole source of carbon and energy. Samples were taken every 12 h for 36 h (in the case of LB medium) and every 24 h for 120 h (in the case of AC medium). The values plotted are the means of three replicates.

Growth rate analysis of strains deprived of the individual megaplasmids was performed in rich LB medium, as well as in AC minimal salt medium. This revealed that the removal of plasmids pAMI1, pAMI4 and pAMI8 had no effect on the growth rate (Figure 2). Interestingly, the pAMI1-less derivative grew in the form of irregular flocks (Additional file 13), which were in fact the aggregations of bacterial cells. In the case of the pAMI6-less strain, growth in LB medium was significantly decreased, and it was completely inhibited in AC medium (Figure 2). This indicates that the presence of plasmid pAMI6 enables growth of this *P. aminophilus* strain in minimal medium.

Based on the obtained results we have classified the pAMI-replicons into two groups: (i) essential (pAMI5, pAMI6) and (ii) non-essential (pAMI1, pAMI2, pAMI3, pAMI4, pAMI7 and pAMI8) genetic elements.

Additionally, to extend the functional analyses, the host ranges of pAMI1-pAMI8 replicons were examined using their plasmid mini-derivatives cloned within plasmid pABW1. The tested strains of *Alphaproteobacteria* were classified into two orders: (i) *Rhodobacterales*, represented by *Paracoccus versutus* UW225 and *Paracoccus pantotrophus* KL100, and (ii) *Rhizobiales*, represented by the families *Brucellaceae* (*Ochrobactrum* sp. LM19R) and *Rhizobiaceae*, including members of the *Rhizobium*/*Agrobacterium* group (*Rhizobium etli* CE3 and *Agrobacterium tumefaciens* LBA288) and the *Sinorhizobium*/*Ensifer* group (*Sinorhizobium* sp. LM21R). Replication abilities of pAMI1-pAMI8 were also tested in *Alcaligenes* sp. LM16R (*Betaproteobacteria*) and *E. coli* BR825 (*Gammaproteobacteria*).

This analysis revealed that mini-derivatives of pAMI1, pAMI2, pAMI3, pAMI4, pAMI7 and pAMI8 could replicate in all analyzed strains of *Alphaproteobacteria*, while the introduction of pAMI5 and pAMI6 into *Sinorhizobium* sp. and *Ochrobactrum* sp. was impossible, which may be due to strong incompatibility with their native replicons. We were also unable to introduce any of the analyzed plasmids into representatives of the *Beta*- or *Gammaproteobacteria* (Additional file 14). These findings indicate the relatively narrow host range of the analyzed replicons, being limited to members of the *Alphaproteobacteria* class.

### Essential genetic elements – chromids

The curing experiments showed that the pAMI5 replicon could not be removed from JCM 7686 cells. Close inspection of the genetic content of this plasmid revealed that 62 (23.5%) of its genes are conserved in the chromosomes of *P. denitrificans* PD1222 (Additional file 11), and that 45 of them are singletons in the JCM 7686 genome. These genes encode several proteins of unknown function, but also proteins that are probably involved in the host metabolism, including (i) iron transport, (ii) D-pantothenate synthesis, (iii) motility, (iv) folate one-carbon metabolism and (v) *de novo* NAD biosynthesis. Interestingly, the *nadABC* (JCM7686_pAMI5p135-137) genes are the only ones encoding components of the *de novo* NAD biosynthetic pathway (Additional file 15) identified within the JCM 7686 genome, which may explain the essential nature of the pAMI5 replicon. It is important to mention that the loss of *nadABC* genes most probably cannot be compensated *via* the NAD salvage pathway, since the predicted NAD pyrophosphatase (an enzyme involved in the process) is also encoded by pAMI5 replicon (JCM7686_pAMI5p033 gene) (the remaining components of this pathway were identified in the JCM 7686 chromosome). Moreover, pAMI5 contains the only copies of (i) the *alkB* gene (JCM7686_pAMI5p075) induced during adaptive responses and involved in the direct reversal of alkylation damage [56] and (ii) the *hslJ* gene (JCM7686_pAMI5p111) encoding a heat shock protein [57].

All the aforementioned genes may play an important role in the JCM 7686 metabolism and we speculate that some of them may be responsible for the pAMI5 essentiality. However this needs to be experimentally confirmed.

Essential element status can also be assigned to the pAMI6 replicon. Although, we were able to remove it from JCM 7686 cells cultivated in LB medium, the growth of the pAMI6-less strain was completely inhibited in minimal salt medium (Figure 2). Analysis of the genetic content of pAMI6 revealed the presence of several modules that are typically encoded on chromosomes. Corresponding genes are involved in histidine, folate, glycerophospholipid, purine, selenoamino acid, sulfur, propanoate, glyoxylate and dicarboxylate metabolism. A surprising finding was the *amtB* gene (JCM7686_pAMI6p067, the only copy of this gene in the JCM 7686 genome), which encodes a NH_4_^+^ transporter of the Amt/Rh family [58]. Presence of this gene on pAMI6 may explain why this plasmid is essential for growth in minimal salt medium, where the only nitrogen source is NH_4_Cl, which cannot be transported into the cell in the absence of an appropriate transporter. This finding was confirmed by the analysis of the strain growth in minimal medium with casamino acids as a nitrogen source instead of ammonium chloride. pAMI6-less strain was able to growth in such medium, but its growth rate was significantly decreased. This suggests that there are also some additional (to *amtB*) genes, which are responsible for the essentiality of this replicon.

A growing number of studies in the field of genomics have produced data suggesting that the structure of many bacterial genomes is more complex than previously assumed. Many bacteria bear additional, large, autonomous replicons, which (like chromosomes) carry a pool of housekeeping genes [59-62]. Unlike typical plasmids, such replicons are necessary for the viability of their hosts, and for this reason they were initially defined as “secondary chromosomes”. However, this name is inadequate since these replicons possess many characteristics typical of plasmids: they contain plasmid-like replication systems and other genetic modules of plasmid origin. Bioinformatic analyses of these replicon sequences indicated that they were generated by the transfer of genetic information from chromosomes to plasmids co-residing in the cell. Due to their dualistic properties, they have been reclassified into a separate, newly distinguished group with properties of both chromosomes and plasmids: the chromids [60]. Our analyses indicate that pAMI5 and pAMI6 can be classified into this group of elements as well.

### Non-essential genetic elements – plasmids

Six of the predicted JCM 7686 plasmids (including the previously described plasmids pAMI2, pAMI3 and pAMI7 [23,25]) were readily removed from the host cells. Among these dispensable replicons are the two *repABC*-type megaplasmids, pAMI4 and pAMI8 (Figure 1). Interestingly, pAMI4 and pAMI8 carry almost as many putative transposase genes (20 and 17, respectively; complete and truncated) as the whole chromosome (25 genes), and many more than the other replicons (0–4 genes) (Table 1). Within pAMI4 and pAMI8 we identified 101 genes encoding predicted transporters involved in the transport of (i) amino acids/dipeptides (*dpp*-like gene clusters), (ii) sn-glycerol-3-phosphate (*ugp*-like gene clusters), (iii) taurine (*tau*-like gene cluster) and (iv) inorganic ions [mainly iron (*fec*-like gene clusters), but also potassium, sodium and various heavy metals]. Genes encoding predicted transporters constitute 21 and 10% of the genetic information carried by plasmids pAMI4 and pAMI8, respectively. Moreover, within pAMI8 we identified a *vir*-gene cluster of a type IV secretion system, which is frequently found in *Alphaproteobacteria* megaplasmids [63], and 5 putative *dsb*-like genes the products of which may be involved in introducing disulfide bonds into diverse substrate proteins [64].

As mentioned above, the removal of pAMI1 from JCM 7686 cells influenced colony morphology and affected the growth mode in rich LB medium (but not in minimal-salt AC medium) (Additional file 13). Analysis of the pAMI1 sequence revealed that 40% of its genes encode proteins involved in amino acid transport and metabolism. Since amino acids serve as the main nitrogen source in LB medium, while nitrogen is provided by ammonium ions in AC medium, this may explain the observed growth differences.

### Prophages and other prophage-related regions

**Figure 3:**
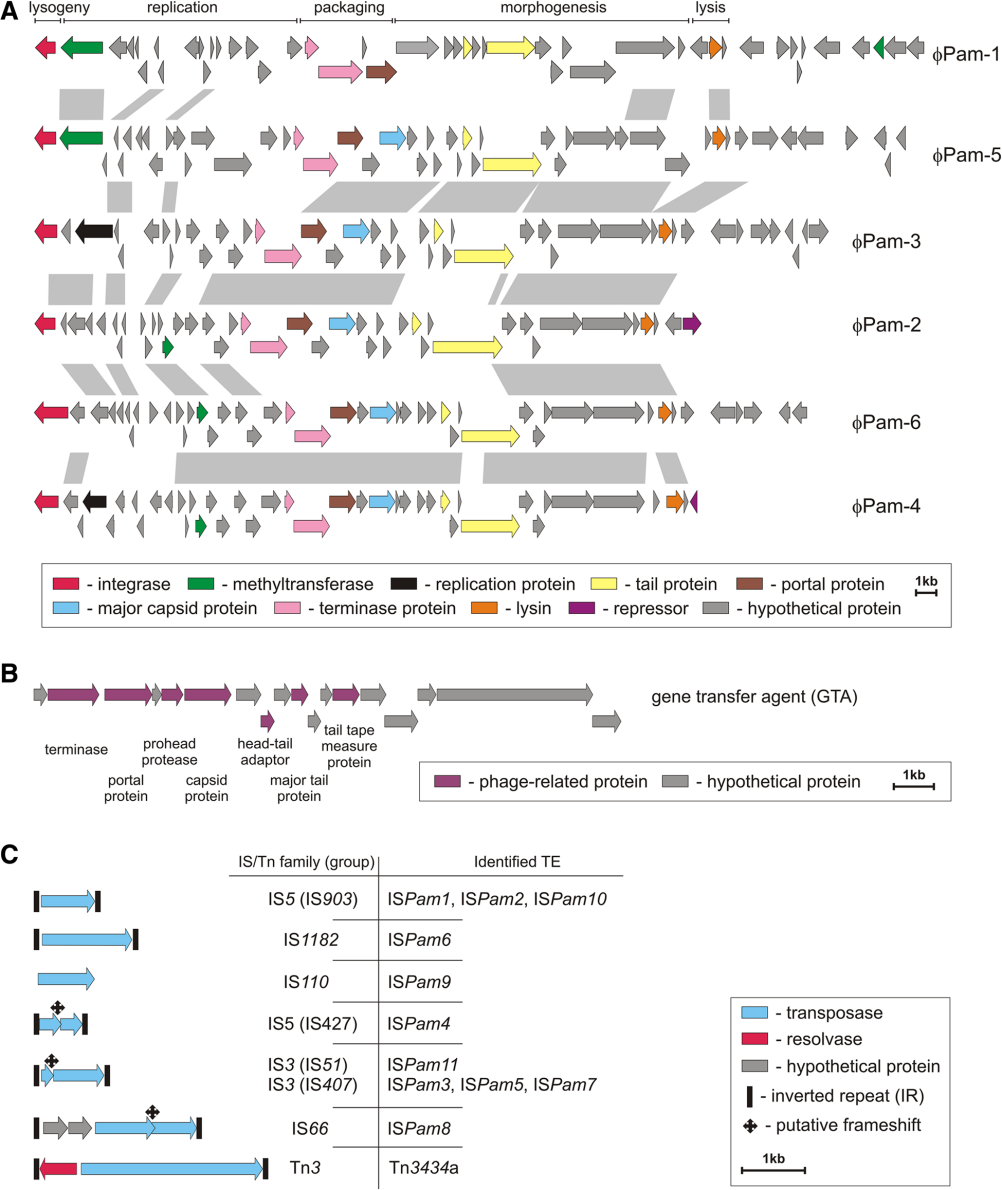
**Prophage-like regions and transposable elements within the *P. aminophilus *JCM 7686 genome.** The putative CDSs, colored according to their category, are shown by arrows representing their direction of transcription. **(A)** Genetic organization and comparison of the prophage-like regions within the *P. aminophilus* genome. Predicted gene clusters contain lysogeny, replication, structural and lysis-associated phage genes. The gray-shaded areas connect regions of prophages sharing ≥95% nucleotide sequence identity. **(B)** Genetic organization of the putative gene transfer agent (GTA) region. **(C)** Genetic organization of the insertion sequences and non-composite transposon Tn*3434*a of *P. aminophilus*. The families and names of the identified TEs are shown in the table. Inverted repeats (IRL – left IR; IRR – right IR) flanking ISs are marked by black vertical bars.

In the *P. aminophilus* chromosome, we identified 10 regions encoding phage-related proteins (Figure 1). These constitute 8.2% of the host chromosome. Close inspection revealed that only 6 of them encode a full set of proteins crucial for the phage “life cycle”. Thus only these regions were considered to represent putative prophages and designated ϕPam-1 to ϕPam-6, respectively (Figure 3A). The predicted sizes of the identified prophages range from 32.9 to 43.9 kb and they comprise between 41 and 49 phage-related genes coding for proteins involved in integration, replication, packaging, capsid and tail assembly, and lysis. For four of the prophages we could identify putative integration sites, which were tRNA sequences (tRNA-Pro for ϕPam-1, tRNA-Arg for ϕPam-3, tRNA-Gly for ϕPam-5 and tRNA-Met for ϕPam-6).

The vast majority of genes annotated within the prophage regions encode hypothetical proteins of unknown function. However, since prophages may encode fitness factors (e.g. virulence genes or metabolic modules) for their lysogenic hosts (e.g. [65]) we performed in-depth homology searches and found that genes JCM7686_0776 and JCM7686_1223 (within ϕPam-1 and ϕPam-2) encode tellurite resistance proteins (TerB). Moreover, we found that all but one (ϕPam-3) of the prophages encode methyltransferases (Figure 3A).

The JCM 7686 prophages exhibit high reciprocal nucleotide sequence similarity (Figure 3A), but they share only limited and localized homology with other prophages. This finding suggests their uniqueness among so far identified bacteriophages. Comparative analyses of the phage structural proteins revealed that five of the JCM 7686 prophages (ϕPam-2-ϕPam-6) encode major capsid proteins similar to those of HK97-like phages (*Siphoviridae*), which may suggest some evolutionary relationship.

To identify functional phages we applied the method of aggressive induction with mitomycin C. Using this procedure, only one of the JCM 7686 prophages, ϕPam-6, was induced. The ϕPam-6 prophage is flanked in the genome by 17-bp direct repeats (5′-CCCTCCTCCGCTACCAT-3′) which were recognized as the phage attachment site. In addition, ϕPam-6 encodes a serine family recombinase, rather than a tyrosine family integrase that is typical for phages (including the other JCM 7686 prophages). Following induction, bacteriophage particles were visualized by transmission electron microscopy using negative staining with uranyl acetate (Additional file 16) and it was confirmed by the restriction analysis that they contain ϕPam-6 DNA. Hence, ϕPam-6 is the first functional phage identified in *Paracoccus* spp.

Among the predicted prophage-related regions we also identified one gene transfer agent (GTA) cluster of 14.7 kb, which contains 18 putative genes (Figure 3B). This GTA cluster is located upstream of the *cysE* gene, encoding serine O-acetyltransferase (involved in cysteine biosynthesis), which is a common location for GTAs in bacterial genomes [66]. The identified region shares synteny and exhibits 80% nucleotide sequence identity with a GTA cluster found within chromosome II of *Paracoccus denitrificans* PD1222 [GenBank:CP000490]. Moreover, the *P. aminophilus* GTA exhibits homology to other RcGTA-like gene clusters (the archetype of this group is *Rhodobacter capsulatus* GTA) identified in various representatives of *Alphaproteobacteria*[66].

### Transposable elements (TEs)

Analysis of the JCM 7686 genome identified 72 predicted transposase genes (Table 1); however, 32 of them are truncated (Additional file 17). The encoded transposases were classified and particular TEs distinguished (Additional file 17).

We identified 11 types of complete insertion sequences representing 5 IS families [IS*3* (IS*407*, IS*51* groups), IS*5* (IS*427*, IS*903* groups), IS*66*, IS*110* and IS*1182*] (Additional file 17). Most of the identified ISs are present as a single copy in the JCM 7686 genome. The exceptions are IS*Pam1* and *ISPam2* (both have 2 copies), as well as IS*Pam5* (8 copies). As shown in Figure 3C, the majority of the identified ISs contain a single ORF encoding a transposase (members of the IS*903* group of the IS*5* family, IS*110* and IS*1182* families) or carry two overlapping ORFs and possess a conserved frame-shift motif (members of the IS*407* group of the IS*3* family and IS*427* group of the IS*5* family). The frame-shift sequences are most probably responsible for the generation of a fusion protein (ORF1 + ORF2) as a result of programmed translational frame-shifting [67]. A putative trans-frame transposase may also be potentially produced by IS*Pam8* of the IS*66* family. IS*Pam8* also contains two additional ORFs (Figure 3C), which may be involved in the regulation of transposition.

Within the plasmid component of the JCM 7686 genome we also identified a non-composite, cryptic transposon Tn*3434a* of the Tn*3* family. It is present in 4 copies: in pAMI1, pAMI7 and pAMI8 (2 copies). The presence of two identical divergently oriented copies of Tn*3434a* within pAMI8 resulted in inversion of an approx. 15-kb DNA region due to site-specific resolvase-mediated recombination.

In a previous study, using a trap plasmid strategy, we showed that IS*Pam1*, IS*Pam2*, IS*Pam3* and Tn*3434a* are fully functional elements [17]. However, such analyses have some limitations because, although it may identify most active elements, other functional TEs can be missed due to their low transposition frequency.

### Comparative genomics of *Paracoccus* spp

Until now, the annotated genome sequences of only four *Paracoccus* spp. strains are available in public databases: *P. denitrificans* PD1222 ([GenBank:CP000489], [GenBank:CP000490] and [GenBank:CP000491]), *Paracoccus* sp. strain TRP [7], *P. denitrificans* SD1 [8] and *Paracoccus* sp. N5 ([GenBank:NZ_AQUO01000001], [GenBank:NZ_AQUO01000002] and [GenBank:NZ_AQUO01000003]). In this study we add the genome of *P. aminophilus* JCM 7686 and comparative analysis of five *Paracoccus* spp. genomes is now possible.

**Figure 4:**
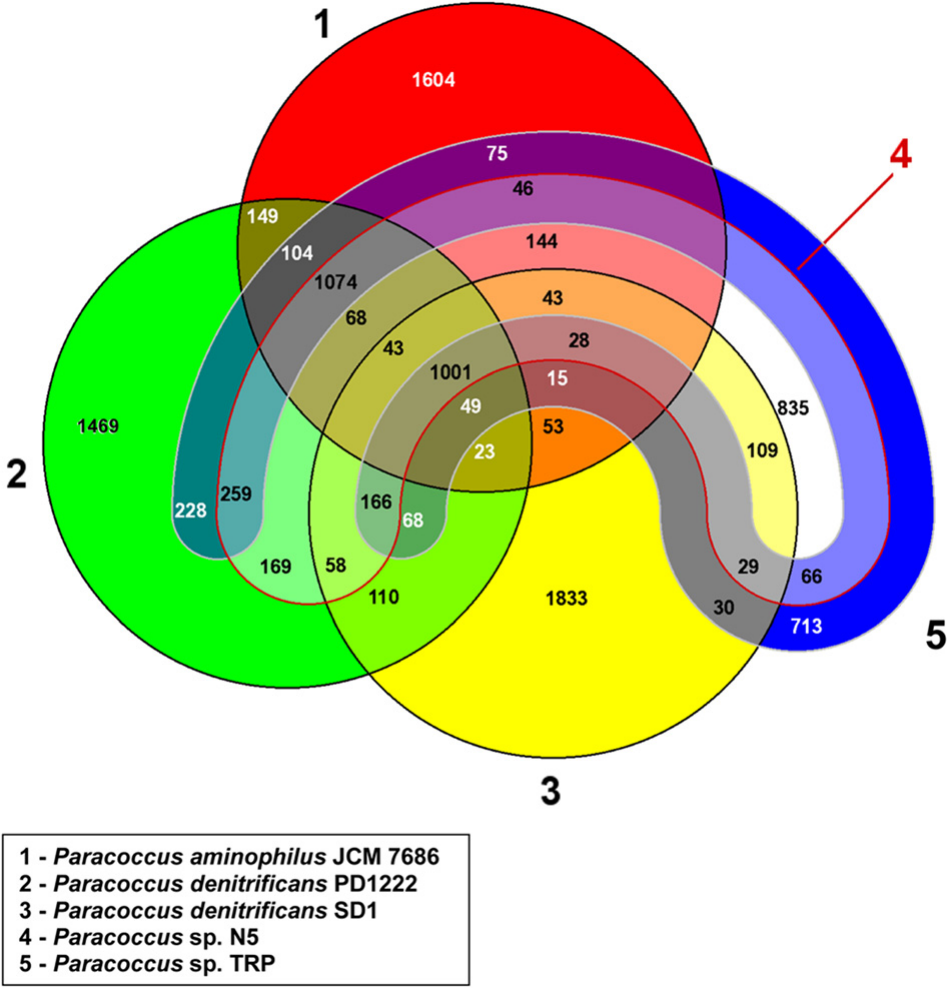
**Venn diagram of the comparison of five sequenced and annotated *Paracoccus *genomes.** The differently colored areas represent the compared strains. The central area describes the core genome consisting of 1001 genes.

A total of 1001 genes (20 - 27% of all genes) can be considered as core *Paracoccus* genes, since they are present in all five analyzed genomes (Figure 4). Functional classification of the proteins encoded by these genes showed that, in a vast majority of cases, they belong to five COG groups (J, O, C, F, H) that represent basic housekeeping functions, expected to be encoded on chromosomes, and therefore conserved among bacterial genomes.

Comparative genomic analyses disclosed some specific features of the *P. aminophilus* genome. The chromosome of strain JCM 7686 contains 757 singletons (22% of all genes) that are mainly located within prophages, although other gene clusters are also included, e.g. a type III secretion system with 19 unique genes. The remaining singleton genes are encoded by the plasmids (46% of all plasmid genes). A relatively large portion of singletons within the genome is a feature of all the analyzed *Paracoccus* genomes, which suggests that these strains are not that closely related.

**Figure 5:**
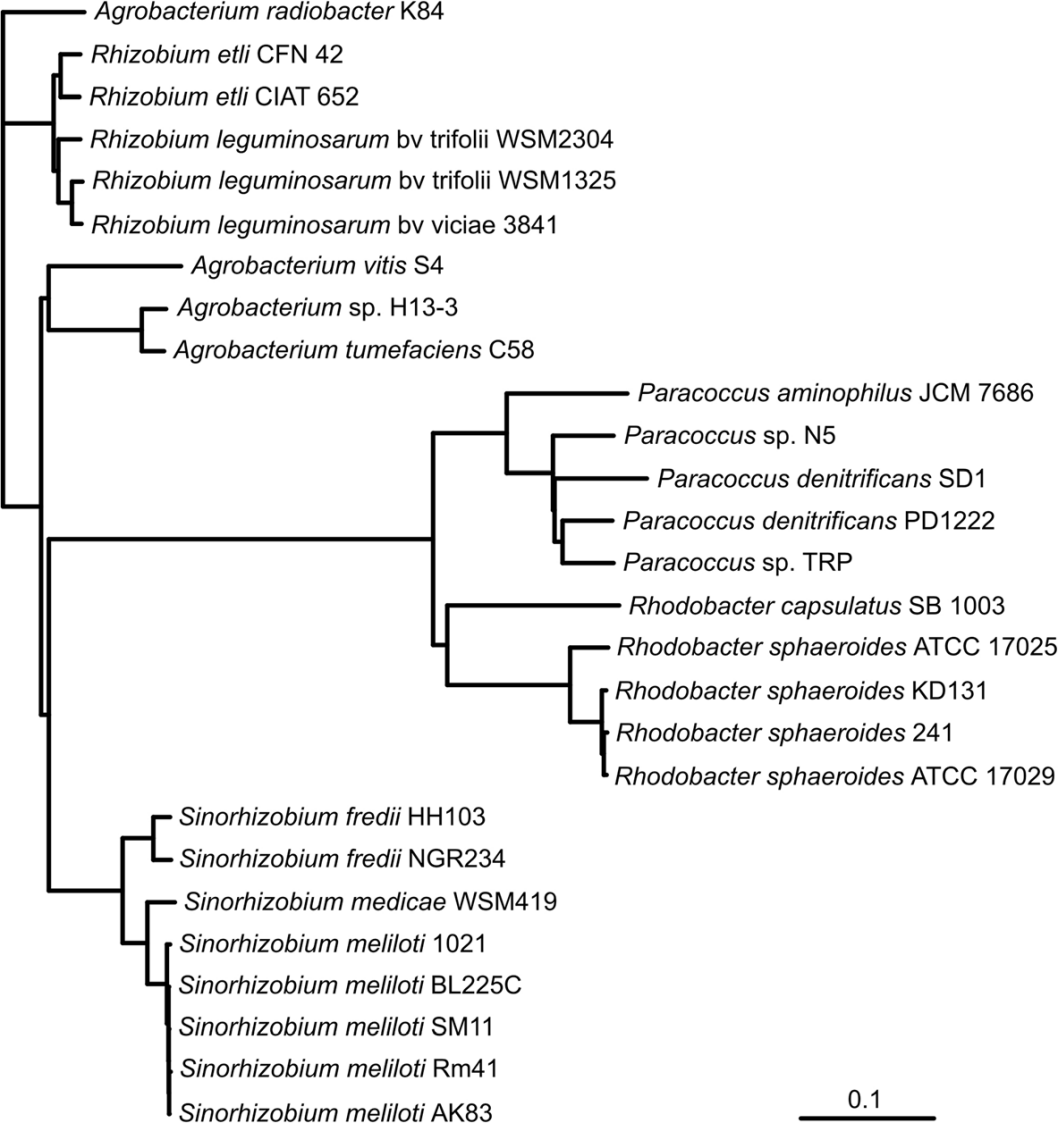
**Phylogenetic tree based on 453 core genes of the selected strains of *Alphaproteobacteria.*** Multiple sequence alignments of concatenated core gene sequences were calculated using EDGAR tool. Plasmid sequences were included.

We also performed a complex phylogenetic analysis of *P. aminophilus* JCM 7686 in relation to other fully sequenced members of the class *Alphaproteobacteria*. A phylogenetic tree based on 453 core genes was computed (Figure 5). *P. aminophilus* JCM 7686 is clustered together with the other completely sequenced *Paracoccus* spp. strains, but it forms an outgroup, which indicates its “distant” relationship to representatives of other species (Figure 5).

## Conclusions

Chromids have been found in bacteria belonging to many different phyla, including *Actinobacteria, Bacteroidetes, Chloroflexi, Cyanobacteria, Deinococcus-Thermus*, *Firmicutes*, *Proteobacteria* and *Spirochaetes*[60,61]. It was shown that chromids of bacteria classified to separate phylogenetic groups carry replication systems of different types, which indicates that these replicons were derived from plasmid precursors typical for particular groups of hosts, and were formed independently in the course of evolution [60,61].

Chromids share a number of major characteristics: (i) considerable size (they are usually the second largest replicons in the cell, being larger than the chromosomes of some bacteria), (ii) the presence of plasmid-type replication systems, (iii) G + C nucleotide contents comparable to that of chromosomes, (iv) codon usage similar to that of chromosomes (points iii and iv indicate long-term co-evolution of chromosomes and chromids), (v) the presence of housekeeping genes, typical for chromosomes (removal of chromids from the bacterial cell causes a lethal effect), and (vi) the presence of adaptive genes, typical for plasmids, enabling adaptation of bacteria to a given ecological niche [60,68,69]. A typical feature of chromids is their high variability, resulting mainly from the low density of housekeeping genes. Therefore, they are considered to be specific “training sites” in which different evolutionary variants are “tested” [68,70,71], which leads to structural variability of multireplicon genomes in different strains of the same genus [72].

The formation of multi-replicon genomes, in which the basic genomic information is split between various replicons, seems to be beneficial to bacteria. The presence of additional replicons enables more rapid duplication of the genetic information, and permits the maintenance of a larger genome while keeping a high rate of cell division. Moreover, it was shown that the frequency of dimer generation by bacterial chromosomes increases exponentially in relation to their size; therefore, the reduction of chromosome size (by dividing the genetic information essential for host viability, into two or more replicons) allows minimization of this phenomenon [73].

Many *Paracoccus* spp. genomes also possess a multi-replicon structure. A good example of such a genome architecture is *P. aminophilus* JCM 7686, which carries one chromosome and eight plasmid-like replicons. Our *in silico* analyses revealed that pAMI1, pAMI4, pAMI5, pAMI6 and pAMI8 carry many genes (also predicted core genes) showing homology to ORFs within *P. denitrificans* PD1222 chromosomes (Additional file 11), thus they may be considered as putative chromids. However, relying only on the bioinformatic studies, the concept of essentiality of particular replicons is highly speculative, thus to define their nature we applied additional analyses.

In this study, acknowledging the importance of functional analyses, we have shown that only pAMI5 and pAMI6 should be classified as chromids, since their presence in the host cells is essential for their proper functioning. pAMI5 could not be removed from the host cells at all, and while it was possible to obtain a pAMI6-less derivative, this strain was unable to grow in minimal media, i.e. in conditions similar to those in the natural environment. Based on this observation we propose the classification of bacterial chromids into two types: “primary” chromids (e.g. pAMI5), which are indispensable for host viability and carry genes of the core genome, thus their elimination from the host cells is impossible under any environmental conditions, and “secondary” chromids (e.g. pAMI6) which were probably formed quite recently from an evolutionary point of view (e.g. they contain different REP systems, and have more characteristics typical for plasmids). Moreover, secondary chromids seems to carry a species (or genera) -specific pool of genes that are crucial for survival in the natural environment, but are not essential under “safe” laboratory conditions, thus the replicons are only “facultatively” essential [74].

According to this definition, the chromosome II of *P. denitrificans* PD1222 should be reclassified as a primary chromid. It is noteworthy that chromosome II of PD1222 and pAMI5 carry related *dnaA*-like replication systems, which may be a typical feature of the primary chromids of *Paracoccus* spp. This may facilitate further classifications of such replicons in other representative of this genus.

## Methods

### Strains, plasmids and culture conditions

The strains used in this study are presented in Additional file 18. They were grown in Luria-Bertani (LB) medium [75], TY medium (*Rhizobium etli* CE3) [76] and the minimal salts medium AC [77] at 37°C (*E. coli*) and 30°C (other strains). Where necessary, the media were supplemented with arabinose (0.2%), glucose (1%), kanamycin (50–1000 μg/ml) and rifampicin (50 μg/ml). The plasmids used and constructed in this study are described in Additional file 19.

### DNA isolation, standard genetic manipulations and introduction of plasmid DNA into bacterial cells

The isolation of total DNA and plasmids, as well as common DNA manipulation methods were performed as described by Sambrook and Russell (2001) [75]. The visualization of mega-sized replicons was achieved by in-gel lysis and DNA electrophoresis according to a method described by Wheatcroft et al. (1990) [78]. PCR was performed in a Mastercycler using HiFi polymerase (Qiagen; with supplied buffer), dNTP mixture, total DNA (or plasmid DNA) of *P. aminophilus* as the template and appropriate oligonucleotide primers (Additional file 20). Transformation of *E. coli* strains was performed according to the method of Kushner [79]. Triparental mating was performed as previously described [80].

### Determination of the sequence specificity of methyltransferases using the endonuclease protection assay

Putative MTase genes were PCR-amplified from *P. aminophilus* genomic DNA using specific primer pairs (Additional file 20). The amplified genes were cloned into expression vectors pET-28a or pET-30a (Additional file 19). Obtained plasmids were used to transform *E. coli* ER2566 and the strains were propagated for expression. To repress T7 RNA polymerase expression in the ER2566 strains before induction, glucose was added to cultures at a final concentration of 1%. The DNAs of recombinant plasmids isolated from IPTG-induced and non-induced bacterial cultures were used as substrates for cleavage by selected restriction enzymes.

### Phage excision and transmission electron microscopy

Mitomycin C (0.5 mg/ml) was added to a logarithmic phase culture of *P. aminophilus* in 20 ml LB medium. Following incubation for a further 8 h, the culture was centrifuged at 11,000 g for 10 min to pellet the cells and the supernatant fraction was passed through a membrane filter (0.22 μm pore size). Phage particles were collected by centrifugation (2 h at 100,000 g) and resuspended in 50 μl of SM buffer (50 mM Tris–HCl, pH 7.5, 100 mM NaCl, 8 mM MgSO_4_ × 7H_2_O, 0.01% gelatin). One drop of the suspension was applied to the surface of a Formvar-coated grid, negatively stained with 2% uranyl acetate [81], and then examined using a LEO 912AB transmission electron microscope.

### DNA sequencing

Total genomic DNA was isolated from an overnight culture of *P. aminophilus* JCM 7686 using the DNeasy Blood & Tissue Kit (Qiagen). This was used for the construction of NGS (Next Generation Sequencing) libraries: (i) GS FLX + shotgun library [using the GS FLX + library preparation kit (Roche)], (ii) an 8-kb-long paired-end library [using the GS FLX Paired-End kit (Roche)] and (iii) Illumina Paired-End library [using the Illumina TruSeq2.0 kit (Illumina Inc.)]. The genomic libraries were sequenced using a Genome Sequencer FLX + System (Roche) and Illumina HiScanSQ Genome Analyzer (Illumina Inc.) in the DNA Sequencing and Oligonucleotide Synthesis Laboratory (oligo.pl) at the Institute of Biochemistry and Biophysics, Polish Academy of Sciences. The sequence data was assembled into contigs and scaffolds using Newbler De Novo Assembler (454 Sequencing System Software, Roche). Any remaining gaps were closed using the Expand Long Template PCR System (Roche), followed by Sanger sequencing with an ABI3500 Genetic Analyzer (Life Technologies) using BigDye Terminator Mix v. 3.1 chemistry (Life Technologies).

### Bioinformatics

Automatic gene prediction and annotation of the JCM 7686 genome were performed using GenDB 2.0 [82]. Automatic annotations and intergenic regions were analyzed and corrected manually by means of BLAST programs [83] and the PRIAM tool [84]. Putative tRNA genes were identified with the tRNAscan-SE program [85]. Each gene was functionally classified by assigning a Cluster of Orthologous Groups (COG) number and its corresponding COG category [86]. Comparison searches for insertion sequences were performed with ISfinder [87]. Finally, the Artemis software was used to visualize the genome [88].

Comparisons between the JCM 7686 genome and the genomes of other representatives of the *Alphaproteobacteria* (i.e. synteny analyses, identification of orthologous genes and classification of genes as core genes or singletons) were performed using the EDGAR tool [89]. It was also used for the creation of the phylogenetic tree. To construct the tree, 453 core genes from 27 genomes of the *Alphaproteobacteria* were computed. The multiple alignments for all core genes were created using MUSCLE [90]. Non matching parts of the alignments were masked using GBLOCKS [91] and removed subsequently. The remaining parts of all alignments were concatenated to one multiple alignment, which then was used to generate the phylogenetic tree applying PHYLIP [92]. Other comparative analyses were performed with the GeneOrder4.0 tool [93].

### Nucleotide sequence accession numbers

The nucleotide sequences of *P. aminophilus* JCM 7686 chromosome and extrachromosomal replicons pAMI1-8 have been annotated and deposited in GenBank (NCBI) with respective accession numbers: CP006650, CP006651, GQ410978, GQ468939, CP006652, CP006653, CP006654, GQ468938, CP006655.

## Abbreviations

CDS: Coding sequence; COG: Cluster of Orthologous Group; DMF: *N,N*-dimethylformamide; GTA: Gene transfer agent; HPK: Histidine protein kinase; IR: Inverted repeat; IS: Insertion sequence; LB: Luria-bertani; MGE: Mobile genetic elements; MTase: methyltransferase; NAD: Nicotinamide adenine dinucleotide; NaMN: Nicotinic acid mononucleotide; NCBI: National Center for Biotechnology Information; ORF: Open reading frame; PCR: Polymerase chain reaction; TE: Transposable element; TMo: Transposable module; Tn: Transposon.

## Competing interests

The authors declare that they have no competing interests.

## Authors’ contributions

LD and DB conceived and designed the experiments; LD coordinated the project; LD, JC, DW, MR, PM and MS performed the experiments; LD, JC, MR and DW analyzed the data; LD, DB, AS and AP contributed reagents/materials/analysis tools; DB, AS and AP supervised the work; LD, DB and DW wrote the paper. All authors read and approved the final manuscript.

## Supplementary Material

Additional file 1 **COG categories of the proteins of *P. aminophilus *** **JCM 7686.** Click here for file

Additional file 2 **Diversity and distribution of 217 transcriptional regulators of *P. aminophilus *** **JCM 7686.** Click here for file

Additional file 3 **Two-component systems and histidine kinases encoded by the *P. aminophilus *** **JCM 7686 genome.** Click here for file

Additional file 4 **Toxin-antitoxin systems encoded by the *P. aminophilus *** **JCM 7686 genome.** Click here for file

Additional file 5 **Chaperones and co-chaperonins encoded by the *P. aminophilus *** **JCM 7686 genome.** Click here for file

Additional file 6 **Genes encoding DNA repair related proteins within the *P. aminophilus *** **JCM 7686 genome.** Click here for file

Additional file 7 **Restriction patterns of *P. aminophilus *** **JCM 7686 genomic DNA cleaved with selected restriction endonucleases showing the protection of GANTC sites by the CcrM methylase (JCM7685_3079).** ND - undigested DNA. M – GeneRuler 100–10,000 bp size marker. Click here for file

Additional file 8 **Summary of the sensitivity of various restriction endonucleases to DNA modifications introduced by the JCM7686_0772 and JCM7686_2655 proteins (m**^**5**^**C MTases).** Click here for file

Additional file 9 **Summary of the sensitivity of various restriction endonucleases to DNA modifications introduced by the JCM7686_0815 protein (m**^**4**^**C MTase).** Click here for file

Additional file 10 **Summary of the sensitivity of various restriction endonucleases to DNA modifications introduced by the JCM7686_1231, JCM7686_2255 and JCM7686_2934 proteins (m**^**6**^**A MTases).** Click here for file

Additional file 11 **The number of homologous proteins encoded by JCM 7686 plasmids and *P. denitrificans *** **PD1222 chromosomes.** The analysis was performed using the GeneOrder 4.0 tool. Proteins were considered homologous only if the BLAT threshold scores were >200. The results were verified manually by BLAST comparisons. Click here for file

Additional file 12 Plasmid profiles of the JCM 7686 wild-type strain and its derivatives deprived of particular megasized-replicons. Click here for file

Additional file 13 The growth mode of wild-type JCM 7686 and the pAMI1-less derivative in liquid LB medium. Click here for file

Additional file 14 **Host ranges of *P. aminophilus *** **JCM 7686 pAMI1-8 replicons.** Click here for file

Additional file 15 **Schematic diagram of *de novo *** **NAD biosynthesis from aspartate to nicotinic acid mononucleotide (NaMN).** Click here for file

Additional file 16 **Transmission electron micrograph of tailed bacteriophage ϕPam-6 of *P. aminophilus *** **JCM 7686.** Click here for file

Additional file 17 **Transposase genes within the *P. aminophilus *** **JCM 7686 genome.** Click here for file

Additional file 18 Bacterial strains used in this study. Click here for file

Additional file 19 Plasmids used and constructed in this study. Click here for file

Additional file 20 Oligonucleotide primers used in this study. Click here for file

## References

[B1] Baker SC Ferguson SJ Ludwig B Page MD Richter OM van Spanning RJ Molecular genetics of the genus *Paracoccus*: metabolically versatile bacteria with bioenergetic flexibility Microbiol Mol Biol Rev 1998 62 4 1046 1078 9841665 10.1128/mmbr.62.4.1046-1078.1998 PMC98939

[B2] Katayama Y Hiraishi A Kuraishi H *Paracoccus thiocyanatus* sp. nov., a new species of thiocyanate-utilizing facultative chemolithotroph, and transfer of *Thiobacillus versutus* to the genus *Paracoccus* as *Paracoccus versutus* comb. nov. with emendation of the genus Microbiology 1995 141 1469 1477 10.1099/13500872-141-6-1469 7545513

[B3] Kelly DP Euzeby JP Goodhew CF Wood AP Redefining *Paracoccus denitrificans* and *Paracoccus pantotrophus* and the case for a reassessment of the strains held by international culture collections Int J Syst Evol Microbiol 2006 56 2495 2500 10.1099/ijs.0.64401-0 17012585

[B4] Kumaraswamy R Sjollema K Kuenen G van Loosdrecht M Muyzer G Nitrate-dependent [Fe(II)EDTA]2- oxidation by *Paracoccus ferrooxidans* sp. nov., isolated from a denitrifying bioreactor Syst Appl Microbiol 2006 29 276 286 10.1016/j.syapm.2005.08.001 16682296

[B5] Doronina NV Trotsenko YA Krausova VI Suzina NE *Paracoccus methylutens* sp. nov. - a new aerobic facultatively methylotrophic bacterium utilizing dichloromethane Syst Appl Microbiol 1998 21 230 236 10.1016/S0723-2020(98)80027-1

[B6] Sun M Luo Y Teng Y Christie P Jia Z Li Z Tenax TA extraction to understand the rate-limiting factors in methyl-beta-cyclodextrin-enhanced bioremediation of PAH-contaminated soil Biodegradation 2013 24 365 375 10.1007/s10532-012-9593-2 23001628

[B7] Li K Wang S Shi Y Qu J Zhai Y Xu L Xu Y Song J Liu L Rahman MA Genome sequence of *Paracoccus* sp. Strain TRP, a chlorpyrifos biodegrader J Bacteriol 2011 193 7 1786 1787 10.1128/JB.00014-11 21257769 PMC3067656

[B8] Siddavattam D Karegoudar TB Mudde SK Kumar N Baddam R Avasthi TS Ahmed N Genome of a novel isolate of *Paracoccus denitrificans* capable of degrading *N,N*-dimethylformamide J Bacteriol 2011 193 19 5598 5599 10.1128/JB.05667-11 21914898 PMC3187436

[B9] Baj J Piechucka E Bartosik D Wlodarczyk M Plasmid occurrence and diversity in the genus *Paracoccus* Acta Microbiol Pol 2000 49 3–4 265 270 11293660

[B10] Bartosik D Putyrski M Dziewit L Malewska E Szymanik M Jagiello E Lukasik J Baj J Transposable modules generated by a single copy of insertion sequence IS*Pme1* and their influence on structure and evolution of natural plasmids of *Paracoccus methylutens* DM12 J Bacteriol 2008 190 9 3306 3313 10.1128/JB.01878-07 18296518 PMC2347374

[B11] Bartosik D Baj J Sochacka M Piechucka E Wlodarczyk M Molecular characterization of functional modules of plasmid pWKS1 of *Paracoccus pantotrophus* DSM 11072 Microbiology 2002 148 Pt 9 2847 2856 12213930 10.1099/00221287-148-9-2847

[B12] Bartosik D Baj J Wlodarczyk M Molecular and functional analysis of pTAV320, a *repABC*-type replicon of the *Paracoccus versutus* composite plasmid pTAV1 Microbiology 1998 144 3149 3157 10.1099/00221287-144-11-3149 9846751

[B13] Bartosik D Witkowska M Baj J Wlodarczyk M Characterization and sequence analysis of the replicator region of the novel plasmid pALC1 from *Paracoccus alcaliphilus* Plasmid 2001 45 3 222 226 10.1006/plas.2000.1505 11407917

[B14] Bartosik D Baj J Bartosik AA Wlodarczyk M Characterization of the replicator region of megaplasmid pTAV3 of *Paracoccus versutus* and search for plasmid-encoded traits Microbiology 2002 148 Pt 3 871 881 11882723 10.1099/00221287-148-3-871

[B15] Bartosik D Sochacka M Baj J Identification and characterization of transposable elements of *Paracoccus pantotrophus* J Bacteriol 2003 185 13 3753 3763 10.1128/JB.185.13.3753-3763.2003 12813068 PMC161580

[B16] Bartosik D Szymanik M Baj J Identification and distribution of insertion sequences of *Paracoccus solventivorans* Appl Environ Microbiol 2003 69 12 7002 7008 10.1128/AEM.69.12.7002-7008.2003 14660342 PMC310034

[B17] Dziewit L Baj J Szuplewska M Maj A Tabin M Czyzkowska A Skrzypczyk G Adamczuk M Sitarek T Stawinski P Insights into the transposable mobilome of *Paracoccus* spp. (*Alphaproteobacteria*) PLoS One 2012 7 2 e32277 10.1371/journal.pone.0032277 22359677 PMC3281130

[B18] Mikosa M Sochacka-Pietal M Baj J Bartosik D Identification of a transposable genomic island of *Paracoccus pantotrophus* DSM 11072 by its transposition to a novel entrapment vector pMMB2 Microbiology 2006 152 Pt 4 1063 1073 16549670 10.1099/mic.0.28603-0

[B19] Szuplewska M Bartosik D Identification of a mosaic transposable element of *Paracoccus marcusii* composed of insertion sequence IS*Pmar4* (IS*As1* family) and an IS*1247a*-driven transposable module (TMo) FEMS Microbiol Lett 2009 292 2 216 221 10.1111/j.1574-6968.2009.01495.x 19187199

[B20] van Spanning RJ de Boer AP Slotboom DJ Reijnders WN Stouthamer AH Isolation and characterization of a novel insertion sequence element, IS*1248*, in *Paracoccus denitrificans* Plasmid 1995 34 1 11 21 10.1006/plas.1995.1029 7480167

[B21] Urakami T Araki H Oyanagi H Suzuki K Komagata K *Paracoccus aminophilus* sp. nov. and *Paracoccus aminovorans* sp. nov., which utilize *N, N*-dimethylformamide Int J Syst Bacteriol 1990 40 3 287 291 10.1099/00207713-40-3-287 2397196

[B22] Dziewit L Jazurek M Drewniak L Baj J Bartosik D The SXT conjugative element and linear prophage N15 encode toxin-antitoxin-stabilizing systems homologous to the *tad-ata* module of the *Paracoccus aminophilus* plasmid pAMI2 J Bacteriol 2007 189 5 1983 1997 10.1128/JB.01610-06 17158670 PMC1855756

[B23] Dziewit L Adamczuk M Szuplewska M Bartosik D DIY series of genetic cassettes useful in construction of versatile vectors specific for *Alphaproteobacteria* J Microbiol Methods 2011 86 2 166 174 10.1016/j.mimet.2011.04.016 21569803

[B24] Dziewit L Kuczkowska K Adamczuk M Radlinska M Bartosik D Functional characterization of the type II PamI restriction-modification system derived from plasmid pAMI7 of *Paracoccus aminophilus* JCM 7686 FEMS Microbiol Lett 2011 324 1 56 63 10.1111/j.1574-6968.2011.02388.x 22092764

[B25] Dziewit L Dmowski M Baj J Bartosik D Plasmid pAMI2 of *Paracoccus aminophilus* JCM 7686 carries *N, N*-dimethylformamide degradation-related genes whose expression is activated by a LuxR family regulator Appl Environ Microbiol 2010 76 6 1861 1869 10.1128/AEM.01926-09 20118371 PMC2837997

[B26] Studholme DJ Buck M The biology of enhancer-dependent transcriptional regulation in bacteria: insights from genome sequences FEMS Microbiol Lett 2000 186 1 9 10.1111/j.1574-6968.2000.tb09074.x 10779705

[B27] Martinez-Antonio A Collado-Vides J Identifying global regulators in transcriptional regulatory networks in bacteria Curr Opin Microbiol 2003 6 482 489 10.1016/j.mib.2003.09.002 14572541

[B28] Maddocks SE Oyston PC Structure and function of the LysR-type transcriptional regulator (LTTR) family proteins Microbiology 2008 154 3609 3623 10.1099/mic.0.2008/022772-0 19047729

[B29] Subramoni S Venturi V LuxR-family ‘solos’: bachelor sensors/regulators of signalling molecules Microbiology 2009 155 Pt 5 1377 1385 19383698 10.1099/mic.0.026849-0

[B30] Wolanin PM Thomason PA Stock JB Histidine protein kinases: key signal transducers outside the animal kingdom Genome Biol 2002 3 10 REVIEWS3013 10.1186/gb-2002-3-10-reviews3013 PMC244915 12372152

[B31] Drepper T Wiethaus J Giaourakis D Gross S Schubert B Vogt M Wiencek Y McEwan AG Masepohl B Cross-talk towards the response regulator NtrC controlling nitrogen metabolism in *Rhodobacter capsulatus* FEMS Microbiol Lett 2006 258 250 256 10.1111/j.1574-6968.2006.00228.x 16640581

[B32] Wang X Vu A Lee K Dahlquist FW CheA-receptor interaction sites in bacterial chemotaxis J Mol Biol 2012 422 282 290 10.1016/j.jmb.2012.05.023 22659323 PMC3418421

[B33] Lamarche MG Wanner BL Crepin S Harel J The phosphate regulon and bacterial virulence: a regulatory network connecting phosphate homeostasis and pathogenesis FEMS Microbiol Rev 2008 32 461 473 10.1111/j.1574-6976.2008.00101.x 18248418

[B34] Ausmees N Jacobs-Wagner C Spatial and temporal control of differentiation and cell cycle progression in *Caulobacter crescentus* Annu Rev Microbiol 2003 57 225 247 10.1146/annurev.micro.57.030502.091006 14527278

[B35] Janausch IG Zientz E Tran QH Kroger A Unden G C4-dicarboxylate carriers and sensors in bacteria Biochim Biophys Acta 2002 1553 39 56 10.1016/S0005-2728(01)00233-X 11803016

[B36] Hughes DT Clarke MB Yamamoto K Rasko DA Sperandio V The QseC adrenergic signaling cascade in Enterohemorrhagic *E. coli* (EHEC) PLoS Pathog 2009 5 e1000553 10.1371/journal.ppat.1000553 19696934 PMC2726761

[B37] Bird TH Du S Bauer CE Autophosphorylation, phosphotransfer, and DNA-binding properties of the RegB/RegA two-component regulatory system in *Rhodobacter capsulatus* J Biol Chem 1999 274 16343 16348 10.1074/jbc.274.23.16343 10347192

[B38] Jung K Veen M Altendorf K K + and ionic strength directly influence the autophosphorylation activity of the putative turgor sensor KdpD of *Escherichia coli* J Biol Chem 2000 275 40142 40147 10.1074/jbc.M008917200 11016946

[B39] Jourlin C Bengrine A Chippaux M Mejean V An unorthodox sensor protein (TorS) mediates the induction of the tor structural genes in response to trimethylamine N-oxide in *Escherichia coli* Mol Microbiol 1996 20 1297 1306 10.1111/j.1365-2958.1996.tb02648.x 8809780

[B40] Boor KJ Bacterial stress responses: what doesn’t kill them can make then stronger PLoS Biol 2006 4 e23 10.1371/journal.pbio.0040023 16535775 PMC1326283

[B41] Butala M Zgur-Bertok D Busby SJ The bacterial LexA transcriptional repressor Cell Mol Life Sci 2009 66 82 93 10.1007/s00018-008-8378-6 18726173 PMC11131485

[B42] Ferullo DJ Lovett ST The stringent response and cell cycle arrest in *Escherichia coli* PLoS Genet 2008 4 e1000300 10.1371/journal.pgen.1000300 19079575 PMC2586660

[B43] Christensen SK Mikkelsen M Pedersen K Gerdes K RelE, a global inhibitor of translation, is activated during nutritional stress Proc Natl Acad Sci U S A 2001 98 14328 14333 10.1073/pnas.251327898 11717402 PMC64681

[B44] Lund PA Microbial molecular chaperones Adv Microb Physiol 2001 44 93 140 11407116 10.1016/s0065-2911(01)44012-4

[B45] Wagner EG Cycling of RNAs on Hfq RNA Biol 2013 10 619 626 10.4161/rna.24044 23466677 PMC3710369

[B46] Chen B Zhong D Monteiro A Comparative genomics and evolution of the HSP90 family of genes across all kingdoms of organisms BMC Genomics 2006 7 156 10.1186/1471-2164-7-156 16780600 PMC1525184

[B47] Martins-Pinheiro M Marques RC Menck CF Genome analysis of DNA repair genes in the alpha proteobacterium *Caulobacter crescentus* BMC Microbiol 2007 7 17 10.1186/1471-2180-7-17 17352799 PMC1839093

[B48] Wigley DB Bacterial DNA repair: recent insights into the mechanism of RecBCD, AddAB and AdnAB Nat Rev Microbiol 2013 11 9 13 23202527 10.1038/nrmicro2917

[B49] Menck CF Shining a light on photolyases Nat Genet 2002 32 338 339 10.1038/ng1102-338 12410227

[B50] Rebeil R Nicholson WL The subunit structure and catalytic mechanism of the *Bacillus subtilis* DNA repair enzyme spore photoproduct lyase Proc Natl Acad Sci U S A 2001 98 9038 9043 10.1073/pnas.161278998 11470912 PMC55369

[B51] Falnes PO Johansen RF Seeberg E AlkB-mediated oxidative demethylation reverses DNA damage in *Escherichia coli* Nature 2002 419 178 182 10.1038/nature01048 12226668

[B52] Marinus MG Casadesus J Roles of DNA adenine methylation in host-pathogen interactions: mismatch repair, transcriptional regulation, and more FEMS Microbiol Rev 2009 33 488 503 10.1111/j.1574-6976.2008.00159.x 19175412 PMC2941194

[B53] Collier J Epigenetic regulation of the bacterial cell cycle Curr Opin Microbiol 2009 12 722 729 10.1016/j.mib.2009.08.005 19783470

[B54] Drozdz M Piekarowicz A Bujnicki JM Radlinska M Novel non-specific DNA adenine methyltransferases Nucleic Acids Res 2012 40 5 2119 2130 10.1093/nar/gkr1039 22102579 PMC3299994

[B55] Bartosik D Bialkowska A Baj J Wlodarczyk M Construction of mobilizable cloning vectors derived from pBGS18 and their application for analysis of replicator region of a pTAV202 mini-derivative of *Paracoccus versutus* pTAV1 plasmid Acta Microbiol Pol 1997 46 4 387 392 9516985

[B56] Falnes PO Rognes T DNA repair by bacterial AlkB proteins Res Microbiol 2003 154 531 538 10.1016/S0923-2508(03)00150-5 14527653

[B57] Lilic M Jovanovic M Jovanovic G Savic DJ Identification of the CysB-regulated gene, *hslJ*, related to the *Escherichia coli* novobiocin resistance phenotype FEMS Microbiol Lett 2003 224 239 246 10.1016/S0378-1097(03)00441-5 12892888

[B58] Tremblay PL Hallenbeck PC Ammonia-induced formation of an AmtB-GlnK complex is not sufficient for nitrogenase regulation in the photosynthetic bacterium *Rhodobacter capsulatus* J Bacteriol 2008 190 1588 1594 10.1128/JB.01643-07 18156251 PMC2258696

[B59] Acosta-Cruz E Wisniewski-Dye F Rouy Z Barbe V Valdes M Mavingui P Insights into the 1.59-Mbp largest plasmid of *Azospirillum brasilense* CBG497 Arch Microbiol 2012 194 725 736 10.1007/s00203-012-0805-2 22481309

[B60] Harrison PW Lower RP Kim NK Young JP Introducing the bacterial ‘chromid’: not a chromosome, not a plasmid Trends Microbiol 2010 18 141 148 10.1016/j.tim.2009.12.010 20080407

[B61] Villasenor T Brom S Davalos A Lozano L Romero D Los Santos AG Housekeeping genes essential for pantothenate biosynthesis are plasmid-encoded in *Rhizobium etli* and *Rhizobium leguminosarum* BMC Microbiol 2011 11 66 10.1186/1471-2180-11-66 21463532 PMC3082293

[B62] Landeta C Davalos A Cevallos MA Geiger O Brom S Romero D Plasmids with a chromosome-like role in rhizobia J Bacteriol 2011 193 1317 1326 10.1128/JB.01184-10 21217003 PMC3067620

[B63] Christie PJ Type IV secretion: the *Agrobacterium* VirB/D4 and related conjugation systems Biochim Biophys Acta 2004 1694 219 234 10.1016/j.bbamcr.2004.02.013 15546668 PMC4845649

[B64] Depuydt M Messens J Collet JF How proteins form disulfide bonds Antioxid Redox Signal 2011 15 49 66 10.1089/ars.2010.3575 20849374

[B65] Tinsley CR Bille E Nassif X Bacteriophages and pathogenicity: more than just providing a toxin? Microbes Infect 2006 8 1365 1371 10.1016/j.micinf.2005.12.013 16698301

[B66] Lang AS Beatty JT Importance of widespread gene transfer agent genes in alpha-proteobacteria Trends Microbiol 2007 15 54 62 10.1016/j.tim.2006.12.001 17184993

[B67] Sekine Y Eisaki N Ohtsubo E Translational control in production of transposase and in transposition of insertion sequence IS*3* J Mol Biol 1994 235 5 1406 1420 10.1006/jmbi.1994.1097 8107082

[B68] Galardini M Pini F Bazzicalupo M Biondi EG Mengoni A Replicon-dependent bacterial genome evolution: the case of *Sinorhizobium meliloti* Genome Biol Evol 2013 5 542 558 10.1093/gbe/evt027 23431003 PMC3622305

[B69] Mazur A Stasiak G Wielbo J Koper P Kubik-Komar A Skorupska A Phenotype profiling of *Rhizobium leguminosarum* bv. trifolii clover nodule isolates reveal their both versatile and specialized metabolic capabilities Arch Microbiol 2013 195 255 267 10.1007/s00203-013-0874-x 23417392 PMC3597991

[B70] Cooper VS Vohr SH Wrocklage SC Hatcher PJ Why genes evolve faster on secondary chromosomes in bacteria PLoS Comput Biol 2010 6 e1000732 10.1371/journal.pcbi.1000732 20369015 PMC2848543

[B71] Morrow JD Cooper VS Evolutionary effects of translocations in bacterial genomes Genome Biol Evol 2012 4 1256 1262 10.1093/gbe/evs099 23160175 PMC3542574

[B72] Mazur A Stasiak G Wielbo J Kubik-Komar A Marek-Kozaczuk M Skorupska A Intragenomic diversity of *Rhizobium leguminosarum* bv. trifolii clover nodule isolates BMC Microbiol 2011 11 123 10.1186/1471-2180-11-123 21619713 PMC3123555

[B73] Val ME Skovgaard O Ducos-Galand M Bland MJ Mazel D Genome engineering in *Vibrio cholerae*: a feasible approach to address biological issues PLoS Genet 2012 8 1 e1002472 10.1371/journal.pgen.1002472 22253612 PMC3257285

[B74] Petersen J Frank O Goker M Pradella S Extrachromosomal, extraordinary and essential–the plasmids of the *Roseobacter* clade Appl Microbiol Biotechnol 2013 97 2805 2815 10.1007/s00253-013-4746-8 23435940

[B75] Sambrook J Russell DW Molecular Cloning: a laboratory manual 2001 New York: Cold Spring Harbor Laboratory Press

[B76] Noel KD Sanchez A Fernandez L Leemans J Cevallos MA *Rhizobium phaseoli* symbiotic mutants with transposon Tn*5* insertions J Bacteriol 1984 158 1 148 155 6325385 10.1128/jb.158.1.148-155.1984 PMC215392

[B77] Wood AP Kelly DP Heterotrophic growth of *Thiobacillus* A2 on sugars and organic acids Arch Microbiol 1977 113 3 257 264 10.1007/BF00492033 879963

[B78] Wheatcroft R McRae GD Miller RW Changes in the *Rhizobium meliloti* genome and the ability to detect supercoiled plasmids during bacteroid development Mol Plant Microbe Interact 1990 3 9 17 10.1094/MPMI-3-009

[B79] Kushner SR Boyer HB, Nicosia S An improved method for transformation of *E. coli* with ColE1 derived plasmids Genetic Engineering 1978 Amsterdam: Elsevier/North-Holland 17 23

[B80] Bartosik D Szymanik M Wysocka E Identification of the partitioning site within the *repABC*-type replicon of the composite *Paracoccus versutus* plasmid pTAV1 J Bacteriol 2001 183 21 6234 6243 10.1128/JB.183.21.6234-6243.2001 11591666 PMC100104

[B81] Ackermann HW Basic phage electron microscopy Methods Mol Biol 2009 501 113 126 10.1007/978-1-60327-164-6_12 19066816

[B82] Meyer F Goesmann A McHardy AC Bartels D Bekel T Clausen J Kalinowski J Linke B Rupp O Giegerich R GenDB - an open source genome annotation system for prokaryote genomes Nucleic Acids Res 2003 31 2187 2195 10.1093/nar/gkg312 12682369 PMC153740

[B83] Altschul SF Madden TL Schaffer AA Zhang J Zhang Z Miller W Lipman DJ Gapped BLAST and PSI-BLAST: a new generation of protein database search programs Nucleic Acids Res 1997 25 17 3389 3402 10.1093/nar/25.17.3389 9254694 PMC146917

[B84] Claudel-Renard C Chevalet C Faraut T Kahn D Enzyme-specific profiles for genome annotation: PRIAM Nucleic Acids Res 2003 31 6633 6639 10.1093/nar/gkg847 14602924 PMC275543

[B85] Lowe TM Eddy SR tRNAscan-SE: a program for improved detection of transfer RNA genes in genomic sequence Nucleic Acids Res 1997 25 955 964 10.1093/nar/25.5.0955 9023104 PMC146525

[B86] Tatusov RL Fedorova ND Jackson JD Jacobs AR Kiryutin B Koonin EV Krylov DM Mazumder R Mekhedov SL Nikolskaya AN The COG database: an updated version includes eukaryotes BMC Bioinformatics 2003 4 41 10.1186/1471-2105-4-41 12969510 PMC222959

[B87] Siguier P Perochon J Lestrade L Mahillon J Chandler M ISfinder: the reference centre for bacterial insertion sequences Nucleic Acids Res 2006 34 D32 D36 10.1093/nar/gkj014 16381877 PMC1347377

[B88] Carver T Berriman M Tivey A Patel C Bohme U Barrell BG Parkhill J Rajandream MA Artemis and ACT: viewing, annotating and comparing sequences stored in a relational database Bioinformatics 2008 24 2672 2676 10.1093/bioinformatics/btn529 18845581 PMC2606163

[B89] Blom J Albaum SP Doppmeier D Puhler A Vorholter FJ Zakrzewski M Goesmann A EDGAR: a software framework for the comparative analysis of prokaryotic genomes BMC Bioinformatics 2009 10 154 10.1186/1471-2105-10-154 19457249 PMC2696450

[B90] Edgar RC MUSCLE: multiple sequence alignment with high accuracy and high throughput Nucleic Acids Res 2004 32 5 1792 1797 10.1093/nar/gkh340 15034147 PMC390337

[B91] Talavera G Castresana J Improvement of phylogenies after removing divergent and ambiguously aligned blocks from protein sequence alignments Syst Biol 2007 56 4 564 577 10.1080/10635150701472164 17654362

[B92] Felsenstein J PHYLIP - phylogeny inference package (version 3.2) Cladistics 1989 5 164 166

[B93] Mahadevan P Seto D Rapid pair-wise synteny analysis of large bacterial genomes using web-based GeneOrder4.0 BMC Res Notes 2010 3 41 10.1186/1756-0500-3-41 20178631 PMC2844394

