# Supplementary material for: Architecture and functions of a multipartite genome of the methylotrophic bacterium Paracoccus aminophilus JCM 7686, containing primary and secondary chromids
Source: BMC Genomics. 2014 Feb 12;15:124. doi: 10.1186/1471-2164-15-124 (PMC3925955; doi:10.1186/1471-2164-15-124)
Supplement: Additional file 1 — COG categories of the proteins of P. aminophilus JCM 7686. [file 1471-2164-15-124-S1.pdf]

TABLE S1. COG categories of the proteins of *P. aminophilus* JCM 7686.

Proteins predicted functions (COG categories)	Chromosome	pAMI1	pAMI2	pAMI3	pAMI4	pAMI5	pAMI6	pAMI7	pAMI8
**L:** DNA replication, recombination and repair	126	4	7	2	17	5	2	5	37
**K:** transcription	202	15	2	1	43	29	17	1	15
**J:** translation, ribosomal structure and biogenesis	163	2	0	0	6	2	1	0	0
**D:** cell division and chromosome partitioning	21	1	1	0	2	1	1	1	3
**O:** posttranslational modification, protein turnover, chaperones	114	0	0	0	2	6	2	0	9
**M:** cell envelope biogenesis, outer membrane	139	2	0	0	10	5	2	0	3
**N:** cell motility and secretion	24	0	0	0	0	4	0	0	1
**V:** defense mechanisms	38	0	0	0	9	4	3	0	5
**P:** inorganic ion transport and metabolism	210	14	0	0	43	38	20	0	5
**T:** signal transduction mechanisms	89	4	0	0	6	14	7	0	8
**U:** intracellular trafficking and secretion	47	0	1	0	1	0	3	1	9
**C:** energy production and conversion	183	5	0	0	23	10	9	0	20
**G:** carbohydrate transport and metabolism	139	3	0	0	31	18	9	0	6
**E:** amino acid transport and metabolism	385	41	0	0	65	52	31	0	1
**F:** nucleotide transport and metabolism	75	2	0	0	4	3	5	0	0
**H:** coenzyme metabolism	124	3	0	0	8	9	8	0	2
**I:** lipid metabolism	105	5	0	0	19	14	10	0	2
**Q:** secondary metabolite biosynthesis, transport and catabolism	82	10	0	0	17	8	4	0	0
**R:** general function prediction only	316	16	0	1	51	28	18	1	17
function unknown (hypothetical protein)	904	8	2	1	33	28	27	5	61
