# Supplementary figures and images for: Architecture and functions of a multipartite genome of the methylotrophic bacterium Paracoccus aminophilus JCM 7686, containing primary and secondary chromids
Source: BMC Genomics. 2014 Feb 12;15:124. doi: 10.1186/1471-2164-15-124 (PMC3925955; doi:10.1186/1471-2164-15-124)

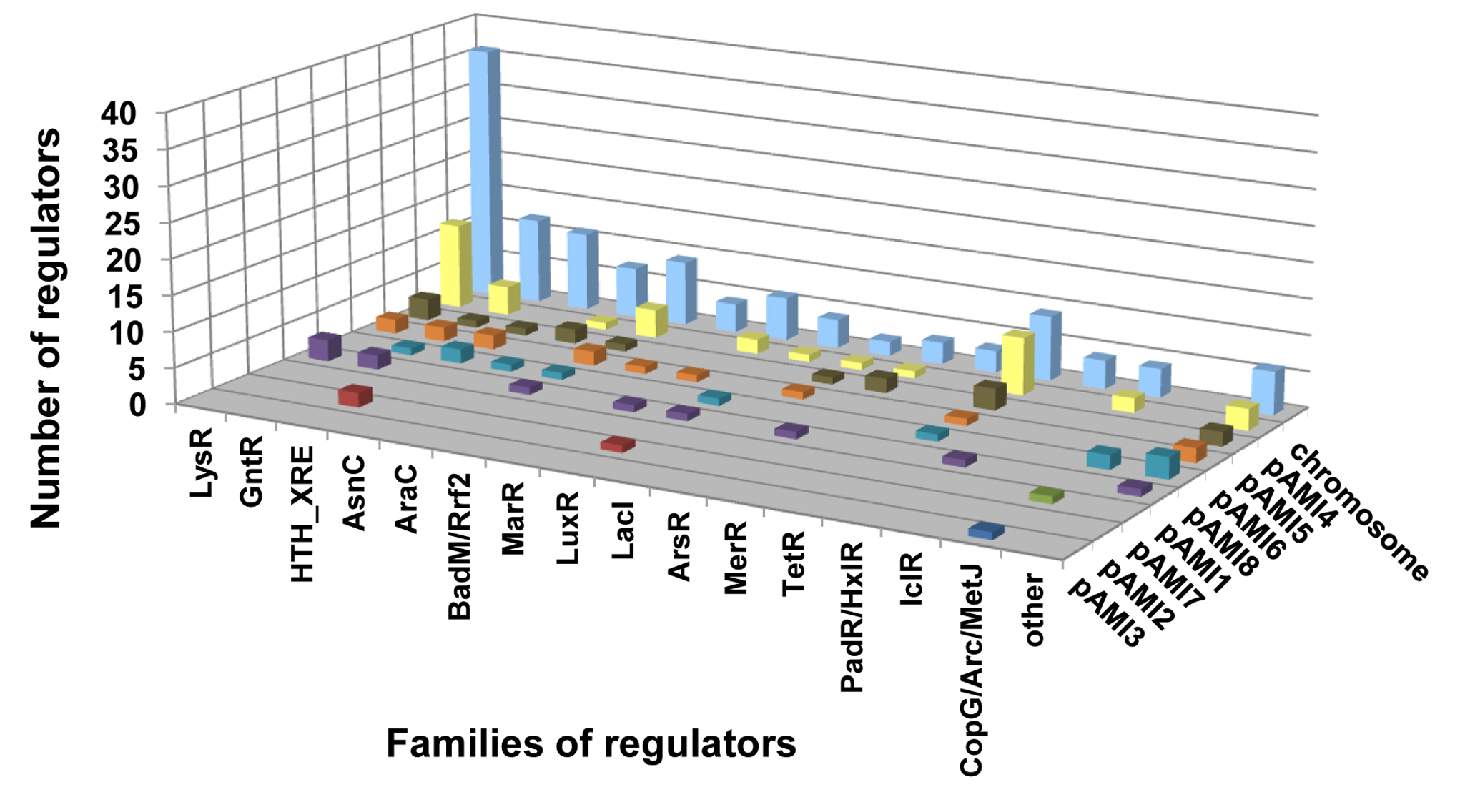

Supplement: Additional file 2 — Diversity and distribution of 217 transcriptional regulators of P. aminophilus JCM 7686. [file 1471-2164-15-124-S2.jpeg]

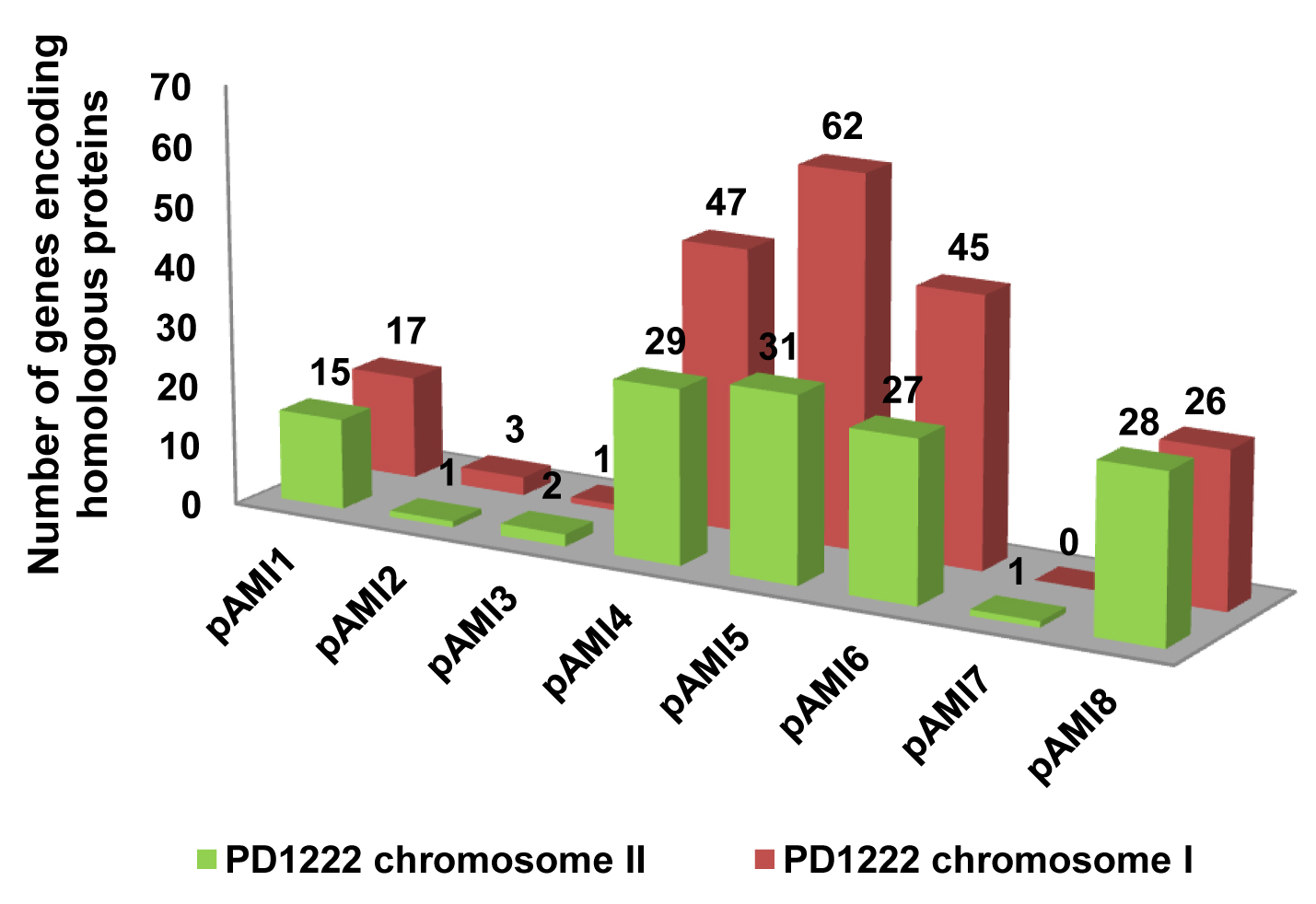

Supplement: Additional file 11 — The number of homologous proteins encoded by JCM 7686 plasmids and P. denitrificans PD1222 chromosomes. The analysis was performed using the GeneOrder 4.0 tool. Proteins were considered homologous only if the BLAT threshold scores were >200. The results were verified manually by BLAST comparisons. [file 1471-2164-15-124-S11.jpeg]

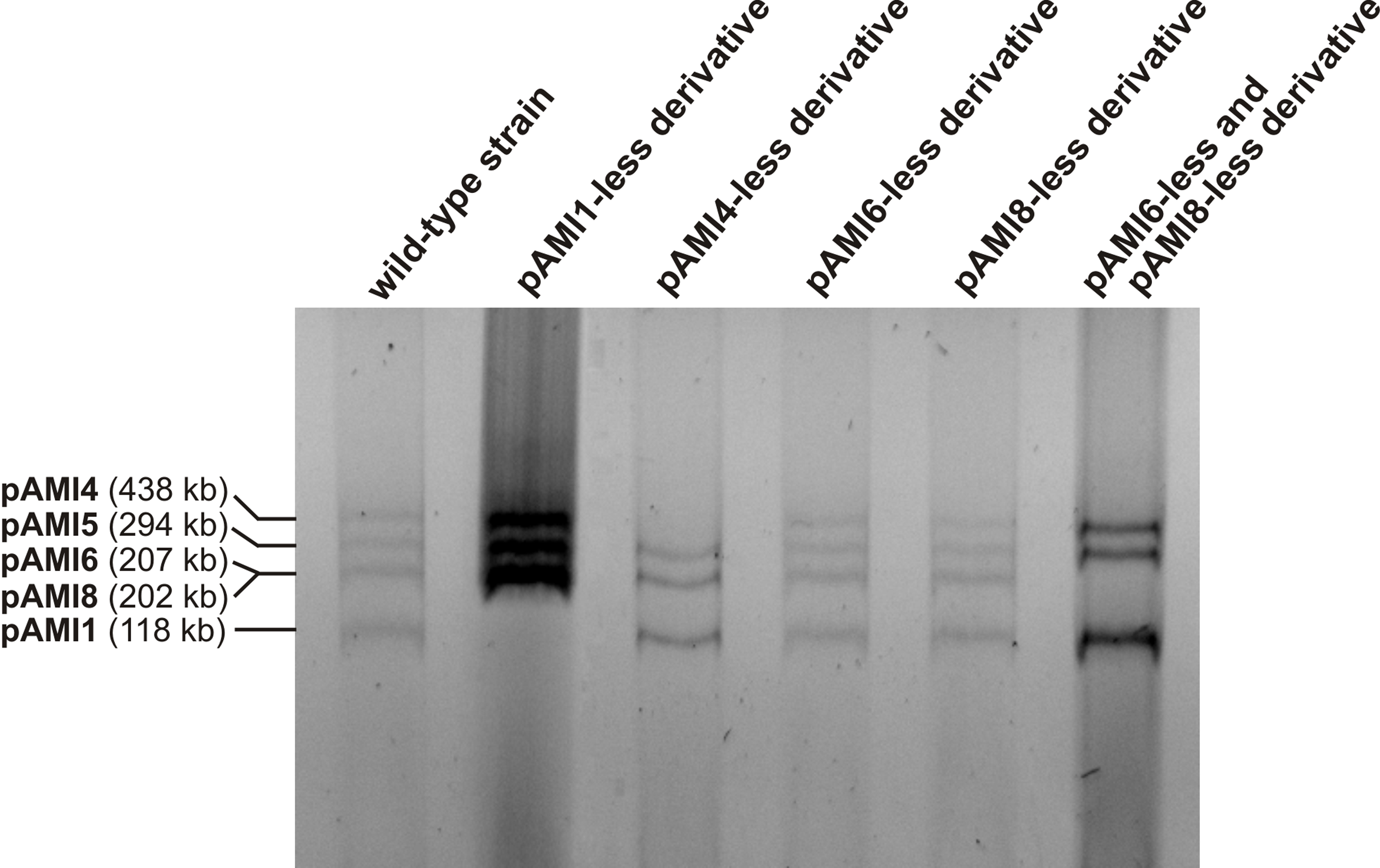

Supplement: Additional file 12 — Plasmid profiles of the JCM 7686 wild-type strain and its derivatives deprived of particular megasized-replicons. [file 1471-2164-15-124-S12.tiff]

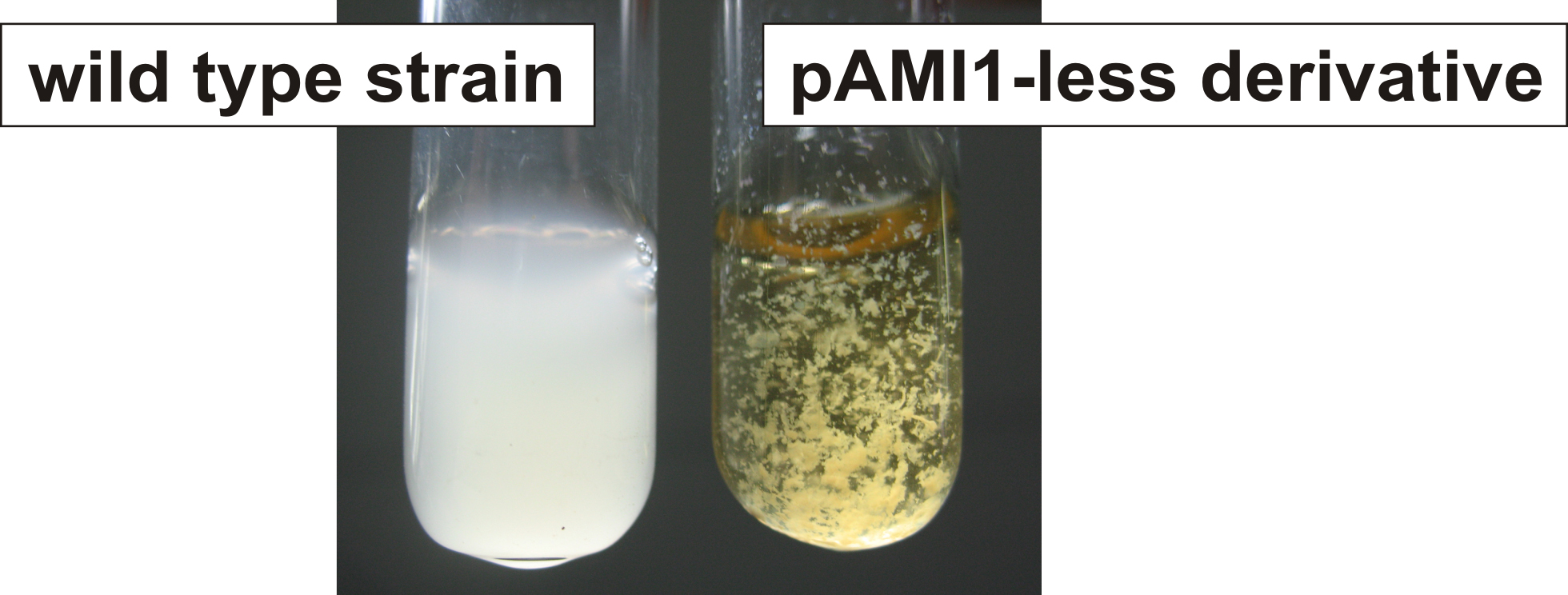

Supplement: Additional file 13 — The growth mode of wild-type JCM 7686 and the pAMI1-less derivative in liquid LB medium. [file 1471-2164-15-124-S13.jpeg]

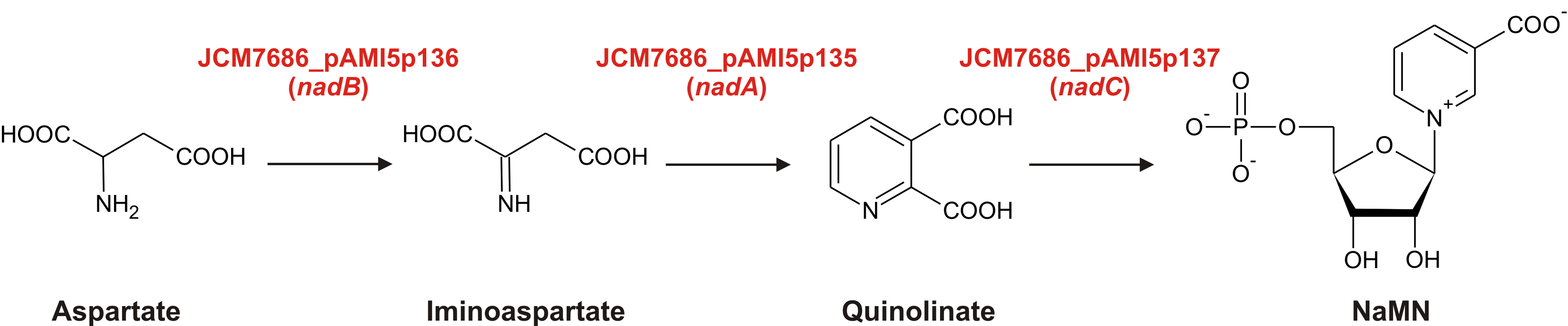

Supplement: Additional file 15 — Schematic diagram of de novo NAD biosynthesis from aspartate to nicotinic acid mononucleotide (NaMN). [file 1471-2164-15-124-S15.tiff]

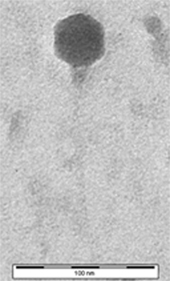

Supplement: Additional file 16 — Transmission electron micrograph of tailed bacteriophage ϕPam-6 of P. aminophilus JCM 7686. [file 1471-2164-15-124-S16.jpeg]
