# Supplementary material for: Architecture and functions of a multipartite genome of the methylotrophic bacterium Paracoccus aminophilus JCM 7686, containing primary and secondary chromids
Source: BMC Genomics. 2014 Feb 12;15:124. doi: 10.1186/1471-2164-15-124 (PMC3925955; doi:10.1186/1471-2164-15-124)
Supplement: Additional file 3 — Two-component systems and histidine kinases encoded by the P. aminophilus JCM 7686 genome. [file 1471-2164-15-124-S3.pdf]

TABLE S2. Two-component systems and histidine kinases encoded by the *P. aminophilus* JCM 7686 genome.

Histidine kinase gene	Response regulator gene	Predicted function (based on the histidine kinase homology)	Replicon
JCM7686_pAMI4p337	JCM7686_pAMI4p336	regulation of a high affinity potassium-uptake	pAMI4
JCM7686_pAMI5p018	JCM7686_pAMI5p019	regulation of a trimethylamine N-oxide reductase respiratory system	pAMI5
JCM7686_pAMI5p061	JCM7686_pAMI5p060	unknown	pAMI5
JCM7686_pAMI5p082	JCM7686_pAMI5p081	unknown	pAMI5
JCM7686_pAMI5p117	JCM7686_pAMI5p118	regulation of expression of virulence factors	pAMI5
JCM7686_pAMI6p023	JCM7686_pAMI6p024	unknown	pAMI6
JCM7686_pAMI6p041	unknown	unknown	pAMI6
JCM7686_pAMI8p124	JCM7686_pAMI8p123	unknown	pAMI8
JCM7686_pAMI8p173	JCM7686_pAMI8p174	unknown	pAMI8
JCM7686_0157	JCM7686_0159	unknown	chromosome
JCM7686_0575	JCM7686_0576	regulation of nitrogen assimilation	chromosome
JCM7686_0577	JCM7686_0578	regulation of nitrogen assimilation	chromosome
JCM7686_0947	unknown	unknown	chromosome
JCM7686_1133	JCM7686_1132	unknown	chromosome
JCM7686_1281	JCM7686_1282	regulation of chemotaxis	chromosome
JCM7686_1314	unknown	unknown	chromosome
JCM7686_1371	JCM7686_1372	unknown	chromosome
JCM7686_1819	JCM7686_1818	unknown	chromosome
JCM7686_1927	JCM7686_1926	unknown	chromosome
JCM7686_2063	unknown	regulation of phosphate homeostasis	chromosome
JCM7686_2280	JCM7686_2279	unknown	chromosome
JCM7686_2539	unknown	regulation of differentiation and cell cycle progression	chromosome
JCM7686_2824	JCM7686_2823	regulation of C4-dicarboxylate metabolism	chromosome
JCM7686_2960	unknown	unknown	chromosome
JCM7686_3261	JCM7686_3260	unknown	chromosome
JCM7686_3369	JCM7686_3370	regulation of expression of virulence factors	chromosome
JCM7686_3383	JCM7686_3385	regulation of methanol and formaldehyde oxidation	chromosome
JCM7686_3423	JCM7686_3425	regulation of several anaerobic processes and assimilation of CO_2 and N_2	chromosome
JCM7686_3465	JCM7686_3468	unknown	chromosome
