# Supplementary material for: Architecture and functions of a multipartite genome of the methylotrophic bacterium Paracoccus aminophilus JCM 7686, containing primary and secondary chromids
Source: BMC Genomics. 2014 Feb 12;15:124. doi: 10.1186/1471-2164-15-124 (PMC3925955; doi:10.1186/1471-2164-15-124)
Supplement: Additional file 4 — Toxin-antitoxin systems encoded by the P. aminophilus JCM 7686 genome. [file 1471-2164-15-124-S4.pdf]

TABLE S3. Toxin-antitoxin systems encoded by the *P. aminophilus* JCM 7686 genome.

Toxin gene	Toxin family	Antitoxin gene	Antitoxin family	Replicon
JCM7686_pAMI2p005	RelE/ParE	JCM7686_pAMI2p006	HTH_XRE	pAMI2
JCM7686_pAMI3p001	RelE/ParE	JCM7686_pAMI3p002	ParD	pAMI3
JCM7686_pAMI4p367	Doc	JCM7686_pAMI4p368	Phd	pAMI4
JCM7686_pAMI5p1216	VapC	JCM7686_pAMI5p215	Phd	pAMI5
JCM7686_pAMI6p005	CcdB	JCM7686_pAMI6p006	CcdA	pAMI6
JCM7686_pAMI7p005	RelE/ParE	JCM7686_pAMI7p004	ParD	pAMI7
JCM7686_pAMI8p005	RelE/ParE	JCM7686_pAMI8p006	ParD	pAMI8
JCM7686_pAMI8p048	VapC	JCM7686_pAMI8p049	Phd	pAMI8
JCM7686_pAMI8p076	RelE/ParE	JCM7686_pAMI8p075	ParD	pAMI8
JCM7686_pAMI8p106	HipA	JCM7686_pAMI8p105	HipB	pAMI8
JCM7686_1676	HipA	JCM7686_1677	HipB	chromosome
