# Supplementary material for: Architecture and functions of a multipartite genome of the methylotrophic bacterium Paracoccus aminophilus JCM 7686, containing primary and secondary chromids
Source: BMC Genomics. 2014 Feb 12;15:124. doi: 10.1186/1471-2164-15-124 (PMC3925955; doi:10.1186/1471-2164-15-124)
Supplement: Additional file 5 — Chaperones and co-chaperonins encoded by the P. aminophilus JCM 7686 genome. [file 1471-2164-15-124-S5.pdf]

TABLE S4. Chaperones and co-chaperonins encoded by the *P. aminophilus* JCM 7686 genome.

Chaperone gene	Homologous protein	Description	Replicon
JCM7686_pAMI4p204	GroEL/Hsp60	major molecular chaperone	pAMI4
JCM7686_pAMI5p111	HslJ	heat shock protein	pAMI5
JCM7686_pAMI5p166	DnaK/Hsp70	major molecular chaperone	pAMI5
JCM7686_pAMI6p154	ClpB-like	ATP-dependent Clp protease ATP-binding subunit	pAMI6
JCM7686_0077	GrpE	heat shock protein	chromosome
JCM7686_0087	ClpP	protease	chromosome
JCM7686_0138	ClpB	ATP-dependent Clp protease ATP-binding subunit	chromosome
JCM7686_0295	Lon	protease	chromosome
JCM7686_0479	FtsH	protease	chromosome
JCM7686_0482	HtpX	heat shock protein, Zn-dependent protease with chaperone function	chromosome
JCM7686_0582	Hfq	RNA chaperone	chromosome
JCM7686_0769	CspB	cold shock protein, RNA chaperone	chromosome
JCM7686_0997	CspA	cold shock protein, RNA chaperone	chromosome
JCM7686_1064	MoxR	MoxR-like ATPase	chromosome
JCM7686_1081	DnaJ-like	DnaJ-class molecular chaperone	chromosome
JCM7686_1391	DegP/HtrA	protease, protein quality control in the periplasm of Gram-negative bacteria	chromosome
JCM7686_1409	Lon	protease	chromosome
JCM7686_1579	ClpX	ATPase with chaperone activity	chromosome
JCM7686_1580	ClpP	protease	chromosome
JCM7686_1874	OmpH	outer membrane chaperone Skp (OmpH)	chromosome
JCM7686_1935	GroES/Hsp10	co-chaperonin	chromosome
JCM7686_1936	GroEL/Hsp60	major molecular chaperone	chromosome
JCM7686_1980	DnaJ-like	DnaJ-class molecular chaperone	chromosome
JCM7686_2155	ClpA	ATPase with chaperone activity	chromosome
JCM7686_2353	IbpA	molecular chaperone	chromosome
JCM7686_2458	CsaA	export-related chaperone	chromosome
JCM7686_2465	Hsp20	small heat shock protein	chromosome
JCM7686_2697	DnaJ-like	DnaJ-class molecular chaperone	chromosome
JCM7686_2977	HslO/Hsp33	disulfide bond chaperones of the HSP33 family	chromosome
JCM7686_3036	DnaK/Hsp70	major molecular chaperone	chromosome
JCM7686_3037	DnaJ	major molecular chaperone	chromosome
JCM7686_3176	ATP12	chaperone required for the assembly of the mitochondrial F1-ATPase	chromosome
JCM7686_3428	SecB	protein-export chaperone	chromosome
JCM7686_3434	HslU/ClpQ	ATP-dependent protease ATP-binding subunit	chromosome
JCM7686_3436	HslV/ClpY	ATP-dependent HslUV protease	chromosome
