# Supplementary material for: Architecture and functions of a multipartite genome of the methylotrophic bacterium Paracoccus aminophilus JCM 7686, containing primary and secondary chromids
Source: BMC Genomics. 2014 Feb 12;15:124. doi: 10.1186/1471-2164-15-124 (PMC3925955; doi:10.1186/1471-2164-15-124)
Supplement: Additional file 6 — Genes encoding DNA repair related proteins within the P. aminophilus JCM 7686 genome. [file 1471-2164-15-124-S6.pdf]

TABLE S5. Genes encoding DNA repair related proteins within the *P. aminophilus* JCM 7686 genome.

Gene	Genes in *E. coli**	Genes in *C. crescentus***	Description	Replicon
**Base excision repair**				
JCM7686_2055	*alkA*	CC_2201	3-methyl-adenine DNA glycosylase II	chromosome
JCM7686_0666	*fpg* (*mutM*)	CC_3707	formamidopyrimidine DNA glycosylase	chromosome
JCM7686_3073	*mutY*	CC_0377	adenine glycosylase	chromosome
JCM7686_0624	*nth*	CC_2272<br>CC_3731	endonuclease III; specific for apurinic and/or apyrimidinic sites	chromosome
JCM7686_0517	*tag*	CC_0382	3-methyl-adenine DNA glycosylase I	chromosome
JCM7686_0757<br>JCM7686_pAMI4p331	*ung* (*udg*)	CC_1333<br>CC_1549<br>CC_2084<br>CC_2333	uracil-DNA glycosylase	chromosome<br>pAMI4
JCM7686_2891<br>JCM7686_3402	*xthA*	CC_2011<br>CC_3706	exodeoxyribonuclease III	chromosome
**Nucleotide excision repair**				
JCM7686_2156	*mfd*	CC_1844	transcription-repair coupling factor	chromosome
JCM7686_2515	*uvrA*	CC_2590	DNA damage recognition protein	chromosome
JCM7686_2310	*uvrB*	CC_2981	DNA damage binding protein	chromosome
JCM7686_3046	*uvrC*	CC_2881	excision nuclease	chromosome
JCM7686_0943	*uvrD*	CC_1528	DNA-dependent ATPase I and helicase II	chromosome
**Mismatch repair**				
JCM7686_3269	*mutL*	CC_0695	DNA mismatch repair protein	chromosome
JCM7686_3143	*mutS*	CC_0012	DNA mismatch repair protein	chromosome
**Direct repair**				
JCM7686_0623<br>JCM7686_pAMI5p076	*ada*	CC_0709<br>CC_3729	O6-methylguanine-DNA methyltransferase; transcription activator/repressor enzyme	chromosome<br>pAMI5
JCM7686_pAMI5p075	*alkB*	CC_0009	1-methyladenine and 3-methylcytosine repair protein	pAMI5
JCM7686_2048	absent	CC_1330	DNA repair photolyase (SplB-like)	chromosome
JCM7686_2677	*ogt*	CC_0659<br>CC_0689	O-6-alkylguanine-DNA/cysteine-protein methyltransferase	chromosome
**Recombinational repair**				
JCM7686_3439	absent	CC_3538	ATP-dependent helicase/nuclease (AddA)	chromosome
JCM7686_3440	absent	CC_3537	ATP-dependent nuclease (AddB)	chromosome
JCM7686_2060	*radA*	CC_1660	DNA repair protein	chromosome
JCM7686_2538	*recA*	CC_1087	DNA strand exchange and renaturation, DNA-dependent ATPase, DNA- and ATP-dependent coprotease	chromosome
JCM7686_0660	*recF*	CC_0159	DNA replication and repair protein	chromosome
JCM7686_2217	*recG*	CC_1437	DNA helicase, resolution of Holliday junctions, branch migration	chromosome
JCM7686_1078	*recJ*	CC_1386	single-stranded-DNA-specific (5'→3') exonuclease	chromosome
JCM7686_0693	*recN*	CC_1983	recombination and repair protein	chromosome
JCM7686_0658	*recO*	CC_1564	recombinational repair protein	chromosome
JCM7686_0328	*recQ*	CC_3465	ATP-dependent DNA helicase	chromosome
JCM7686_1942	*recR*	CC_0269	recombination and repair protein	chromosome

JCM7686_0483	*ruvA*	CC_3237	Holliday junction helicase subunit A; branch migration	chromosome
JCM7686_0481	*ruvB*	CC_1283<br>CC_3236	Holliday junction helicase subunit B; branch migration	chromosome
JCM7686_0484	*ruvC*	CC_3238	Holliday junction nuclease; resolution of structures	chromosome
JCM7686_0280	*sbcB*	absent	exodeoxyribonuclease I	chromosome
JCM7686_2161<br>JCM7686_pAMI8p047	*ssb*	CC_1468	ssDNA-binding protein	Chromosome<br>pAMI8
**Other repair related proteins**				
JCM7686_1991<br>JCM7686_2612<br>JCM7686_2906	*dinB*<br>(*polIV*)	CC_2466	polymerase IV (damage-inducible protein B)	chromosome
JCM7686_1318<br>JCM7686_1972<br>JCM7686_pAMI8p132<br>JCM7686_pAMI8p203	*dnaE*	CC_1926<br>CC_3211	subunit alpha of DNA polymerase III	chromosome<br>pAMI8
JCM7686_2872	*dut*	CC_3713	dUTPase	chromosome
JCM7686_0753	*lexA*	CC_1902	regulator for SOS regulon	chromosome
JCM7686_2218	*ligA*	CC_1522	DNA ligase NAD-dependent	chromosome
JCM7686_pAMI4p364	absent	CC_3610	DNA ligase ATP-dependent	pAMI4
JCM7686_pAMI4p365	*lhr*	CC_2040	Lhr-like helicases	pAMI4
JCM7686_0135<br>JCM7686_2983<br>JCM7686_2998<br>JCM7686_pAMI5p033	*mutT*	CC_0266<br>CC_0407<br>CC_0418<br>CC_0833<br>CC_1126<br>CC_1346<br>CC_1554<br>CC_2652<br>CC_3382<br>CC_3440<br>CC_3650	mutT/NUDIX family hydrolase	chromosome<br>pAMI5
JCM7686_3038<br>JCM7686_pAMI8p117	*radC*	CC_2680<br>CC_2744	DNA repair protein	chromosome<br>pAMI8
JCM7686_pAMI8p201	absent	CC_3213	ImuA repair protein	pAMI8
JCM7686_1973<br>JCM7686_pAMI8p202	absent	CC_3212	ImuB, Y-family DNA polymerase	chromosome<br>pAMI8

* representative of *Gammaproteobacteria*
** representative of *Alphaproteobacteria*
