# Supplementary material for: Architecture and functions of a multipartite genome of the methylotrophic bacterium Paracoccus aminophilus JCM 7686, containing primary and secondary chromids
Source: BMC Genomics. 2014 Feb 12;15:124. doi: 10.1186/1471-2164-15-124 (PMC3925955; doi:10.1186/1471-2164-15-124)
Supplement: Additional file 7 — Restriction patterns of P. aminophilus JCM 7686 genomic DNA cleaved with selected restriction endonucleases showing the protection of GANTC sites by the CcrM methylase (JCM7685_3079). ND - undigested DNA. M – GeneRuler 100–10,000 bp size marker. [file 1471-2164-15-124-S7.pdf]

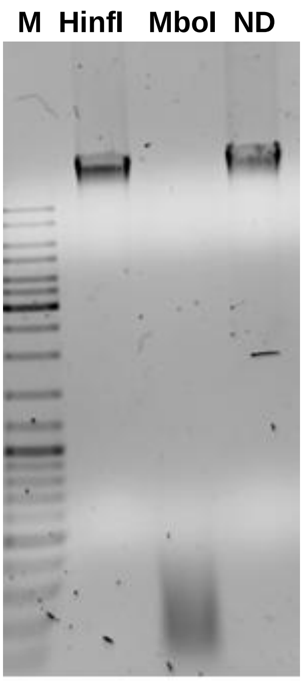

HinfI – recognizes and cleaves the sequence 5’-GANTC-3’
MboI – recognizes and cleaves the sequence 5’-GATC-3’
For the analysis also other restriction endonucleases were used.

Inability to cleave GANTC sequences within *P. aminophilus* JCM 7686 genomic DNA shows that the sequences were methylated (and therefore protected) by CcrM methylase. Analogous analysis was performed with the cloned JCM7685_3079 gene in an *in vitro* experiment.
