# Supplementary material for: Architecture and functions of a multipartite genome of the methylotrophic bacterium Paracoccus aminophilus JCM 7686, containing primary and secondary chromids
Source: BMC Genomics. 2014 Feb 12;15:124. doi: 10.1186/1471-2164-15-124 (PMC3925955; doi:10.1186/1471-2164-15-124)
Supplement: Additional file 8 — Summary of the sensitivity of various restriction endonucleases to DNA modifications introduced by the JCM7686_0772 and JCM7686_2655 proteins (m 5 C MTases). [file 1471-2164-15-124-S8.pdf]

TABLE S6. Summary of the sensitivity of various restriction endonucleases to DNA modifications introduced by the JCM7686_0772 and JCM7686_2655 proteins (m^5C MTases).

REase	Recognition site	Sensitivity to m^5C	Number of sites in pET28_JCM7686_0772	Number of sites in pET28_JCM7686_2655	Cleavage
**AluI**	AG**C**T	S	29	29	P, L
**BglI**	GCcNNNNNGG**C**	S	3	3	P, L
**Bme1390I**	CCNGG	U	32	31	P
**BsuRI**	GG**C**C	S	51	52	P, L
**CaiI**	CAGNNN**C**TG	S	4	4	P, L
**EcoO109I**	RGGN**CC**Y	S	4	5	P
**EcoRII**	C**C**WGG	S	12	12	P, L
**Hin6I**	G**C**GC	S	84	91	P
**HpaII**	**CC**GG	S	43	44	P
**HpyF10VI**	GCNNNNNNNG**C**	S	80	80	P
**MspI**	**C**cGG	S	43	44	P
**NciI**	C**C**SGG	S	20	19	P, L
**PauI**	GCGCGC	U	8	10	P
**PvuII**	CAG**C**TG	S	4	6	P, L
**TaiI**	A**C**GT	S	16	17	P
**XhoI**	CTCGAG	U	3	2	P, L
**Bsh1236I**	**C**G**C**G	S	75	72	Y
**Bsp68I**	TCGCGA	U	3	3	Y
**Bsp143I**	GAT**C**	S	40	43	Y
**BspLI**	GGNNC**C**	S	31	32	Y
**Eco130I**	CCWWGG	U	6	6	Y
**FspBI**	CTAG	U	7	7	Y
**Hin1II**	CATG	U	41	41	Y
**HinfI**	GANT**C**	S	20	21	Y
**MbiI**	CCGCT**C**	S	7	6	Y
**MluI**	ACG**C**GT	S	1	1	Y
**NcoI**	**C**CATGG	S	3	2	Y
**PamDI**	**C**CATGG	S	3	2	Y

Y – complete cleavage; P – partial digestion; L – partial plasmid cleavage; S – sensitive to m^5C; U – unknown sensitivity to m^5C; **C** – the enzyme will not cleave if the **marked** cytosine is methylated; c – the enzyme is not sensitive to methylation of the marked cytosine; C – there are no data on the sensitivity of the enzyme to methylation of the unmarked cytosine.
