# Supplementary material for: Architecture and functions of a multipartite genome of the methylotrophic bacterium Paracoccus aminophilus JCM 7686, containing primary and secondary chromids
Source: BMC Genomics. 2014 Feb 12;15:124. doi: 10.1186/1471-2164-15-124 (PMC3925955; doi:10.1186/1471-2164-15-124)
Supplement: Additional file 9 — Summary of the sensitivity of various restriction endonucleases to DNA modifications introduced by the JCM7686_0815 protein (m 4 C MTase). [file 1471-2164-15-124-S9.pdf]

TABLE S7. Summary of the sensitivity of various restriction endonucleases to DNA modifications introduced by the JCM7686_0815 protein (m^4C MTase).

REase	Recognition site	Sensitivity to m^4C	Number of sites in pET30_JCM7686_0815	Cleavage
**CfrI**	YGGCCR	U	5	P, L
**HaeIII**	GGC**C**	S	26	P*
**Hin6I**	GCGC	U	53	Y
**HpaII**	**CC**GG	S	34	Y
**MspI**	**C**cGG	S	34	Y

* the larger fused DNA fragments were observed only in case of incomplete cleavage of BsuRI sites that overlapped CfrI target sequences (Y**GGCC**R).

Y – complete cleavage; P – partial digestion; L – partial plasmid cleavage; S – sensitive to m^4C; U – unknown sensitivity to m^4C; **C** – the enzyme will not cleave if the **marked** cytosine is methylated; c – the enzyme is not sensitive to methylation of the marked cytosine; C – there are no data on the sensitivity of the enzyme to methylation of the unmarked cytosine.
