# Supplementary material for: Architecture and functions of a multipartite genome of the methylotrophic bacterium Paracoccus aminophilus JCM 7686, containing primary and secondary chromids
Source: BMC Genomics. 2014 Feb 12;15:124. doi: 10.1186/1471-2164-15-124 (PMC3925955; doi:10.1186/1471-2164-15-124)
Supplement: Additional file 10 — Summary of the sensitivity of various restriction endonucleases to DNA modifications introduced by the JCM7686_1231, JCM7686_2255 and JCM7686_2934 proteins (m 6 A MTases). [file 1471-2164-15-124-S10.pdf]

TABLE S8. Summary of the sensitivity of various restriction endonucleases to DNA modifications introduced by the JCM7686_1231, JCM7686_2255 and JCM7686_2934 proteins (m^6A MTases).

REase	Recognition site	Sensitivity to m^6A	Number of sites in pET28_JCM7686_2934	Number of sites in pET28_JCM7686_2255	Number of sites in pET28_JCM7686_1231	Cleavage
**HinfI**	G**A**NTC	S	19	19	18	N
**AluI**	**A**GCT	S	25	25	22	Y
**CseI**	G**A**CGC	S	14	14	13	Y
**NlaIII**	C**A**TG	U	32	32	30	Y
**TaiI**	**A**CGT	S	14	14	14	Y
**TasI**	**A**ATT	U	23	23	21	Y
**Tru1I**	TTA**A**	U	25	25	25	Y

Y – complete cleavage; N – no digestion; S – sensitive to m^6A; U – unknown sensitivity to m^6A; **A** – the enzyme will not cleave if the **marked** adenine is methylated; A – there are no data on the sensitivity of the enzyme to methylation of the unmarked adenine.
