# Supplementary material for: Architecture and functions of a multipartite genome of the methylotrophic bacterium Paracoccus aminophilus JCM 7686, containing primary and secondary chromids
Source: BMC Genomics. 2014 Feb 12;15:124. doi: 10.1186/1471-2164-15-124 (PMC3925955; doi:10.1186/1471-2164-15-124)
Supplement: Additional file 14 — Host ranges of P. aminophilus JCM 7686 pAMI1-8 replicons. [file 1471-2164-15-124-S14.pdf]

TABLE S9. Host ranges of *P. aminophilus* JCM 7686 pAMI1-8 replicons.

Host / Plasmid	pAMI1	pAMI2	pAMI3	pAMI4	pAMI5	pAMI6	pAMI7	pAMI8
Alphaproteobacteria								
Agrobacterium tumefaciens LBA288	+	+	+	+	+	+	+	+
Ochrobactrum sp. LM19R	+	+	+	+	-	-	+	+
Paracoccus versutus UW225	+	+	+	+	+	+	+	+
Paracoccus pantotrophus KL100	+	+	+	+	+	+	+	+
Rhizobium etli CE3	+	+	+	+	+	+	+	+
Sinorhizobium sp. LM 21R	+	+	+	+	-	-	+	+
Betaproteobacteria								
Alcaligenes sp. LM16R	-	-	-	-	-	-	-	-
Gammaproteobacteria								
Escherichia coli BR825	-	-	-	-	-	-	-	-

- unable to replicate
+ able to replicate
