# Supplementary material for: Architecture and functions of a multipartite genome of the methylotrophic bacterium Paracoccus aminophilus JCM 7686, containing primary and secondary chromids
Source: BMC Genomics. 2014 Feb 12;15:124. doi: 10.1186/1471-2164-15-124 (PMC3925955; doi:10.1186/1471-2164-15-124)
Supplement: Additional file 17 — Transposase genes within the P. aminophilus JCM 7686 genome. [file 1471-2164-15-124-S17.pdf]

TABLE S10. Transposase genes within the *P. aminophilus* JCM 7686 genome.

Transposase gene	Truncated/complete	IS name/ Tn name	IS family (IS group)/ Tn family	IS length/ Tn length	Insertion site of complete TEs	Replicon
JCM7686_pAMI1p053	complete	Tn*3434a*	Tn*3*	3695	putrescine transport system permease (*potB*) gene	pAMI1
JCM7686_pAMI2p013	complete	IS*Pam3*	IS*3* (IS*407*)	1197	gene encoding hypothetical protein	pAMI2
JCM7686_pAMI2p014	complete	IS*Pam3*	IS*3* (IS*407*)	1197	gene encoding hypothetical protein	pAMI2
JCM7686_pAMI2p018	complete	IS*Pam4*	IS*5* (IS*427*)	865	intergenic region	pAMI2
JCM7686_pAMI2p019	complete	IS*Pam4*	IS*5* (IS*427*)	865	intergenic region	pAMI2
JCM7686_pAMI4p209	complete	IS*Pam1*	IS*5* (IS*903*)	1050	intergenic region	pAMI4
JCM7686_pAMI4p225	complete	IS*Pam5*	IS*3* (IS*407*)	1197	intergenic region	pAMI4
JCM7686_pAMI4p226	complete	IS*Pam5*	IS*3* (IS*407*)	1197	intergenic region	pAMI4
JCM7686_pAMI4p304	truncated	N/A	IS*1182*	N/A	N/A	pAMI4
JCM7686_pAMI4p305	truncated	N/A	IS*21*	N/A	N/A	pAMI4
JCM7686_pAMI4p306	complete	IS*Pam5*	IS*3* (IS*407*)	1197	intergenic region	pAMI4
JCM7686_pAMI4p307	complete	IS*Pam5*	IS*3* (IS*407*)	1197	intergenic region	pAMI4
JCM7686_pAMI4p333	truncated	N/A	IS*5* (IS*427*)	N/A	N/A	pAMI4
JCM7686_pAMI4p334	complete	IS*Pam5*	IS*3* (IS*407*)	1197	transposase gene	pAMI4
JCM7686_pAMI4p335	complete	IS*Pam5*	IS*3* (IS*407*)	1197	transposase gene	pAMI4
JCM7686_pAMI4p348	truncated	N/A	IS*3* (IS*150*)	N/A	N/A	pAMI4
JCM7686_pAMI4p349	truncated	N/A	IS*3* (IS*150*)	N/A	N/A	pAMI4
JCM7686_pAMI4p350	truncated	N/A	IS*3* (IS*150*)	N/A	N/A	pAMI4
JCM7686_pAMI4p353	truncated	N/A	IS*5* (IS*903*)	N/A	N/A	pAMI4
JCM7686_pAMI4p354	truncated	N/A	IS*5* (IS*903*)	N/A	N/A	pAMI4
JCM7686_pAMI4p357	truncated	N/A	IS*256*	N/A	N/A	pAMI4
JCM7686_pAMI4p358	truncated	N/A	IS*3* (IS*51*)	N/A	N/A	pAMI4
JCM7686_pAMI4p359	complete	IS*Pam5*	IS*3* (IS*407*)	1197	transposase gene	pAMI4
JCM7686_pAMI4p360	complete	IS*Pam5*	IS*3* (IS*407*)	1197	transposase gene	pAMI4
JCM7686_pAMI4p361	complete	IS*Pam6*	IS*1182*	1657	intergenic region	pAMI4
JCM7686_pAMI5p071	truncated	N/A	IS*5* (IS*903*)	N/A	N/A	pAMI5
JCM7686_pAMI5p092	complete	IS*Pam2*	IS*5* (IS*903*)	1054	ABC-type nitrate/sulfonate/bicarbonate transport system, ATPase gene	pAMI5
JCM7686_pAMI5p247	truncated	N/A	IS*3* (IS*407*)	N/A	N/A	pAMI5
JCM7686_pAMI5p248	truncated	N/A	IS*3* (IS*407*)	N/A	N/A	pAMI5
JCM7686_pAMI7p010	complete	Tn*3434a*	Tn*3*	3695	gene encoding hypothetical protein	pAMI7
JCM7686_pAMI8p011	complete	IS*Pam2*	IS*5* (IS*903*)	1054	DNA/RNA helicase (superfamily II, SNF2 family) gene	pAMI8
JCM7686_pAMI8p022	complete	IS*Pam7*	IS*3* (IS*407*)	1271	intergenic region	pAMI8
JCM7686_pAMI8p023	complete	IS*Pam7*	IS*3* (IS*407*)	1271	intergenic region	pAMI8
JCM7686_pAMI8p024	truncated	N/A	IS*L3*	N/A	N/A	pAMI8

JCM7686_pAMI8p025 JCM7686_pAMI8p026	complete (frameshift)	IS*Pam8*	IS*66*	2669	transposase gene	pAMI8
JCM7686_pAMI8p090	complete	N/A	IS*3* (IS*150*)	N/A	N/A	pAMI8
JCM7686_pAMI8p091	truncated					
JCM7686_pAMI8p167	complete	Tn*3434a*	Tn*3*	3695	Na(+)-translocating NADH-quinone reductase subunit C gene	pAMI8
JCM7686_pAMI8p168	complete	IS*Pam1*	IS*5* (IS*903*)	1050	intergenic region	pAMI8
JCM7686_pAMI8p175	truncated	N/A	IS*21*	N/A	N/A	pAMI8
JCM7686_pAMI8p176	truncated	N/A	IS*21*	N/A	N/A	pAMI8
JCM7686_pAMI8p183	complete	Tn*3434a*	Tn*3*	3695	sodium/glutamate symporter gene	pAMI8
JCM7686_pAMI8p196	truncated	N/A	IS*481*	N/A	N/A	pAMI8
JCM7686_pAMI8p197	complete	IS*Pam9*	IS*110*	N/A*	transposase gene	pAMI8
JCM7686_pAMI8p198	truncated	N/A	IS*3* (IS*51*)	N/A	N/A	pAMI8
JCM7686_pAMI8p199	complete	IS*Pam10*	IS*5* (IS*903*)	1054	transposase gene	pAMI8
JCM7686_0276	complete	IS*Pam5*	IS*3* (IS*407*)	1197	transposase gene	chromosome
JCM7686_0277	complete					
JCM7686_0278	truncated	N/A	IS*3* (IS*407*)	N/A	N/A	chromosome
JCM7686_0279	truncated	N/A	IS*3* (IS*407*)	N/A	N/A	chromosome
JCM7686_0292	complete	IS*Pam5*	IS*3* (IS*407*)	1197	gene encoding hypothetical protein	chromosome
JCM7686_0293	complete					
JCM7686_1270	truncated	N/A	IS*3* (IS*51*)	N/A	N/A	chromosome
JCM7686_1274	truncated	N/A	IS*66*	N/A	N/A	chromosome
JCM7686_1275	truncated					
JCM7686_1679	truncated	N/A	IS*66*	N/A	N/A	chromosome
JCM7686_1868	truncated	N/A	IS*3* (IS*407*)	N/A	N/A	chromosome
JCM7686_1869	truncated					
JCM7686_2162	complete	IS*Pam5*	IS*3* (IS*407*)	1197	intergenic region	chromosome
JCM7686_2163	complete					
JCM7686_2224	truncated	N/A	IS*3* (IS*407*)	N/A	N/A	chromosome
JCM7686_2225	truncated					
JCM7686_2291	complete	N/A	Mu-type	N/A	N/A	chromosome
JCM7686_2292	complete					
JCM7686_2296	complete	IS*Pam11*	IS*3* (IS*51*)	1219	transposase gene	chromosome
JCM7686_2297	complete					
JCM7686_2298	truncated	N/A	IS*5* (IS*427*)	N/A	N/A	chromosome
JCM7686_2387	complete	IS*Pam5*	IS*3* (IS*407*)	1197	intergenic region	chromosome
JCM7686_2388	complete					
JCM7686_2390	truncated	N/A	IS*21*	N/A	N/A	chromosome
JCM7686_2391	truncated					

* Since ISs of the IS*110* family do not have IRs, and do not generate DRs, it is not possible to determine the length of IS*Pam9*.
