# Supplementary material for: Architecture and functions of a multipartite genome of the methylotrophic bacterium Paracoccus aminophilus JCM 7686, containing primary and secondary chromids
Source: BMC Genomics. 2014 Feb 12;15:124. doi: 10.1186/1471-2164-15-124 (PMC3925955; doi:10.1186/1471-2164-15-124)
Supplement: Additional file 18 — Bacterial strains used in this study. [file 1471-2164-15-124-S18.pdf]

Table S11. Bacterial strains used in this study.

Strain	Characteristics	Reference
Agrobacterium tumefaciens LBA288	Rif^r	[1]
Alcaligenes sp. LM16R	Rif^r derivative of wild-type strain LM16	[2]
Escherichia coli BR825	*polA::Tn10 trp*	[3]
E. coli ER2566	F^- λ^- *fhuA2* (*lon*) *ompT lacZ::T7 geneI gal sulA11 Δ(mcrC mrr)114::IS10 R*(*mcr-73*::*mini-Tn10)2 R*(*zgb-210::*Tn*10)1* (Tet^S) *endA1* (*Dcm*)	Laboratory collection
E. coli TG1	F' [*traD36 proAB*$^+$ *lacI*^{q} *lacZΔM15*]*supE thi-1 Δ(lac-proAB) Δ(mcrB-hsdSM)5*, ($r_K^- m_K^-$)	Laboratory collection
Ochrobactrum sp. LM19R	Rif^r derivative of wild-type strain LM19	[2]
Paracoccus aminophilus JCM 7686	wild type	[4]
P. aminophilus JCM 7686R	Rif^r derivative of wild-type strain JCM 7686	[5]
P. aminophilus AMI1	pAMI1-less derivative of the strain JCM 7686R	This work
P. aminophilus AMI4	pAMI4-less derivative of the strain JCM 7686R	This work
P. aminophilus AMI6	pAMI6-less derivative of the strain JCM 7686R	This work
P. aminophilus AMI8	pAMI8-less derivative of the strain JCM 7686R	This work
P. aminophilus AMI68	pAMI6-less and pAMI8-less derivative of the strain JCM 7686R	This work
Paracoccus pantotrophus KL100	Rif^r derivative of wild-type strain DSM 11073	[6]
Paracoccus versutus UW225	Rif^r derivative of wild-type strain UW1	[7]
Rhizobium etli CE3	Str^r	[8]
Sinorhizobium sp. LM 21R	Rif^r derivative of wild-type strain LM21	[2]

References

1. Hooykaas PJ, den Dulk-Ras H, Schilperoort RA: **Molecular mechanism of Ti plasmid mobilization by R plasmids: isolation of Ti plasmids with transposon-insertions in *Agrobacterium tumefaciens*.** *Plasmid* 1980, **4**:64-75.
2. Dziewit L, Adamczuk M, Szuplewska M, Bartosik D: **DIY series of genetic cassettes useful in construction of versatile vectors specific for Alphaproteobacteria**. *J Microbiol Methods* 2011, **86**(2):166-174.
3. Ludtke DN, Eichorn BG, Austin SJ: **Plasmid-partition functions of the P7 prophage**. *J Mol Biol* 1989, **209**(3):393-406.
4. Urakami T, Araki H, Oyanagi H, Suzuki K, Komagata K: ***Paracoccus aminophilus* sp. nov. and *Paracoccus aminovorans* sp. nov., which utilize *N,N*-dimethylformamide**. *Int J Syst Bacteriol* 1990, **40**(3):287-291.
5. Dziewit L, Dmowski M, Baj J, Bartosik D: **Plasmid pAMI2 of *Paracoccus aminophilus* JCM 7686 carries *N,N*-dimethylformamide degradation-related genes whose expression is activated by a LuxR family regulator**. *Appl Environ Microbiol* 2010, **76**(6):1861-1869.
6. Bartosik D, Baj J, Bartosik AA, Wlodarczyk M: **Characterization of the replicator region of megaplasmid pTAV3 of *Paracoccus versutus* and search for plasmid-encoded traits**. *Microbiology (Reading, England)* 2002, **148**(Pt 3):871-881.
7. Bartosik D, Baj J, Plasota M, Piechucka E, Wlodarczyk M: **Analysis of *Thiobacillus versutus* pTAV1 plasmid functions.** *Acta Microbiol Pol* 1993, **39**:5-11.
8. Noel KD, Sanchez A, Fernandez L, Leemans J, Cevallos MA: ***Rhizobium phaseoli* symbiotic mutants with transposon Tn5 insertions**. *J Bacteriol* 1984, **158**(1):148-155.
