# Supplementary material for: Architecture and functions of a multipartite genome of the methylotrophic bacterium Paracoccus aminophilus JCM 7686, containing primary and secondary chromids
Source: BMC Genomics. 2014 Feb 12;15:124. doi: 10.1186/1471-2164-15-124 (PMC3925955; doi:10.1186/1471-2164-15-124)
Supplement: Additional file 19 — Plasmids used and constructed in this study. [file 1471-2164-15-124-S19.pdf]

Table S12. Plasmids used and constructed in this study.

Plasmid	Characteristics	Reference
pABW1	Km^r; *ori* pMB1; mobilizable cloning vector; *oriT* RK2	[1]
pABW1-AMI1	Km^r; *ori* pMB1; pABW1 derivative carrying 6.8-kb BamHI restriction fragment of pAMI1 (contains REP and PAR modules)	This work
pABW1-AMI4	Km^r; *ori* pMB1; pABW1 derivative carrying 5.6-kb SalI/KpnI restriction fragment of pAMI4 (contains REP module)	This work
pABW1-AMI5	Km^r; *ori* pMB1; pABW1 derivative carrying 4.0-kb DNA region (contains REP and PAR modules) of pAMI5 (amplified by PCR with primers LAMI5 and RAMI5) inserted between XbaI and SphI sites	This work
pABW1-AMI6	Km^r; *ori* pMB1; pABW1 derivative carrying 1.9-kb DNA region (contains REP module) of pAMI6 (amplified by PCR with primers LAMI6 and RAMI62) inserted between XbaI and SphI sites	This work
pABW1-AMI8	Km^r; *ori* pMB1; pABW1 derivative carrying 4.3-kb DNA region (contains REP and PAR modules) of pAMI8 (amplified by PCR with primers LAMI8 and RAMI8) inserted into EcoRI site	This work
pBBR1MCS-2	Km^r; *ori* pBBR1; broad-host-range cloning vector; mobilizable cloning vector, *oriT* RK2	[2]
pBBR-CcrM	Km^r; *ori* pBBR1; pBBR1MCS-2 derivative carrying 1.4-kb DNA region (contains JCM7686_3079 gene) amplified by PCR with primers FCCRMXB and RCCRMXH and inserted between XbaI and XhoI sites	This work
pET28_JCM7686_1231	Km^r; *ori* pMB1; pET-28a derivative carrying 0.6-kb DNA region (contains JCM7686_1231 gene) amplified by PCR with primers JCM7686_1231F and JCM7686_1231R and inserted between NdeI and SalI sites	This work
pET28_JCM7686_2255	Km^r; *ori* pMB1; pET-28a derivative carrying 0.6-kb DNA region (contains JCM7686_2255 gene) amplified by PCR with primers JCM7686_2255F and JCM7686_2255R	This work
pET28_JCM7686_2655	Km^r; *ori* pMB1; pET-28a derivative carrying 2.1-kb DNA region (contains JCM7686_2655 gene) amplified by PCR with primers JCM7686_2655F and JCM7686_2655R and inserted between NheI and HindIII sites	This work
pET28_JCM7686_2934	Km^r; *ori* pMB1; pET-28a derivative carrying 0.6-kb DNA region (contains JCM7686_2934 gene) amplified by PCR with primers JCM7686_2934F and JCM7686_2934R and inserted between NcoI and SalI sites	This work
pET28_JCM7686_0772	Km^r; *ori* pMB1; pET-28a derivative carrying 2.1-kb DNA region (contains JCM7686_0772 gene) amplified by PCR with primers JCM7686_0772F and JCM7686_0772R and inserted between NheI and HindIII sites	This work
pET-28a	Km^r; *ori* pMB1; expression vector with T7lac promoter	Novagen
pET30_JCM7686_3079	Km^r; *ori* pMB1; pET-30a derivative carrying 1.2-kb DNA region (contains JCM7686_3079 gene) amplified by PCR with primers JCM7686_3079F and JCM7686_3079R and inserted between NdeI and XhoI sites	This work
pET30_JCM7686_0815	Km^r; *ori* pMB1; pET-30a derivative carrying 0.5-kb DNA region (contains JCM7686_0815 gene) amplified by PCR with primers JCM7686_0815F and JCM7686_0815R and inserted between NdeI and XhoI sites	This work
pET-30a	Km^r; *ori* pMB1; expression vector with T7lac promoter	Novagen
pRK2013	Km^r; helper plasmid carrying genes for conjugal transfer of RK2	[3]

References

1. Bartosik D, Bialkowska A, Baj J, Wlodarczyk M: **Construction of mobilizable cloning vectors derived from pBGS18 and their application for analysis of replicator region of a pTAV202 mini-derivative of *Paracoccus versutus* pTAV1 plasmid**. *Acta Microbiol Pol* 1997, **46**(4):387-392.
2. Kovach ME, Phillips RW, Elzer PH, Roop RM, 2nd, Peterson KM: **pBBR1MCS: a broad-host-range cloning vector**. *BioTechniques* 1994, **16**(5):800-802.
3. Ditta G, Stanfield S, Corbin D, Helinski DR: **Broad host range DNA cloning system for gram-negative bacteria: construction of a gene bank of Rhizobium meliloti**. *Proc Natl Acad Sci USA* 1980, **77**(12):7347-7351.
