# Supplementary material for: Architecture and functions of a multipartite genome of the methylotrophic bacterium Paracoccus aminophilus JCM 7686, containing primary and secondary chromids
Source: BMC Genomics. 2014 Feb 12;15:124. doi: 10.1186/1471-2164-15-124 (PMC3925955; doi:10.1186/1471-2164-15-124)
Supplement: Additional file 20 — Oligonucleotide primers used in this study. [file 1471-2164-15-124-S20.pdf]

Table S13. Oligonucleotide primers used in this study.

Primer	Sequence (5'->3')	Function
JCM7686_0772F	GTTGCTAGCATGACCTATCAGCCGCACCTGCC	amplification of JCM7686_0772
JCM7686_0772R	GTTAAGCTTTCACCCAACCCTCCTGTCTTCCTTGATG	amplification of JCM7686_0772
JCM7686_1231F	GTTCATATGGGTAAGCGATCGAATTTCC	amplification of JCM7686_1231
JCM7686_1231R	GTTTCAGTCGACTGCCGCTGCCCTCCCGAAG	amplification of JCM7686_1231
JCM7686_2255F	CAGCGTCGTATTGGCGGGCTATC	amplification of JCM7686_2255
JCM7686_2255R	GACCGAGAGCGGAATCGCTCATG	amplification of JCM7686_2255
JCM7686_2655F	GTTGCTAGCATGACCTATCAACCGCACCTGCC	amplification of JCM7686_2655
JCM7686_2655R	GTTAAGCTTTCACCCAACCCTCCTGTTTTCCTC	amplification of JCM7686_2655
JCM7686_2934F	GTTCCATGGGTAAGCGATCGAATTTCC	amplification of JCM7686_2934
JCM7686_2934R	GTTGTCGACTGCCGCGGCCCTCCCGATAAATTC	amplification of JCM7686_2934
JCM7686_0815F	GTTCATATGAGCGAAACTCCTGTGCTCGATG	amplification of JCM7686_0815
JCM7686_0815R	GTTTTACTCGAGTCGGGTGGCCTCCGGCTTCATG	amplification of JCM7686_0815
JCM7686_3079F	GTTCAT ATGAGGCAGAGCAAAATGAAAACA	amplification of JCM7686_3079
JCM7686_3079R	GTTTCACTCGAG GTTCGGGCGCTGTGCGCCGTCG	amplification of JCM7686_3079
FCCRMXB	GATCTAGAAAGTGTTGCGCGACAGACTC	amplification of JCM7686_3079
RCCRMXH	TACTCGAGCGGCGAAACGAATGGATCAG	amplification of JCM7686_3079
LAMI5	GCTCTAGATGGCGCAAGAAGATCGAAGG	amplification of a replication module of pAMI5
RAMI5	TAGCATGCGCGAAGCGGATTAACCATCG	amplification of a replication module of pAMI5
LAMI6	GACTCTAGAATCCGCTTCGGTCTTCTGG	amplification of a replication module of pAMI6
RAMI62	TAGCATGCGCGTTCTGCGAGATCCTGTC	amplification of a replication module of pAMI6
LAMI8	AGACCTGCAGGACTTCCACTGAATAGCAG	amplification of a replication module of pAMI8
RAMI8	CGAGAATCCACAGAAGCAGCTTGGTCATG	amplification of a replication module of pAMI8
